# Anti-depressant effect of Naringenin-loaded hybridized nanoparticles in diabetic rats via PPARγ/NLRP3 pathway

**DOI:** 10.1038/s41598-024-62676-x

**Published:** 2024-06-12

**Authors:** Salma A. El-Marasy, Mona M. AbouSamra, Passant E. Moustafa, Hoda B. Mabrok, Omar A. Ahmed-Farid, Asmaa F. Galal, Hadir Farouk

**Affiliations:** 1 Department of Pharmacology, Medical Research and Clinical Studies Institute, National Research Centre, Giza, Egypt; 2 Pharmaceutical Technology Department, Pharmaceutical Drug Industries Research Institute, National Research Centre, Giza, Egypt; 3 Nutrition and Food Science Department, Food Industries and Nutrition Research Institute, National Research Centre, Giza, Egypt; 4 Department of Physiology, Egyptian Drug Authority, Giza, Egypt; 5 Narcotics, Ergogenics and Poisons Department, Medical Research and Clinical Studies Institute, National Research Centre, Giza, Egypt

**Keywords:** Biochemistry, Nanoscience and technology, Experimental models of disease

## Abstract

Naringenin (NAR) has various biological activities but low bioavailability. The current study examines the effect of Naringenin-loaded hybridized nanoparticles (NAR-HNPs) and NAR on depression induced by streptozotocin (STZ) in rats. NAR-HNPs formula with the highest in vitro NAR released profile, lowest polydispersity index value (0.21 ± 0.02), highest entrapment efficiency (98.7 ± 2.01%), as well as an acceptable particle size and zeta potential of 415.2 ± 9.54 nm and 52.8 ± 1.04 mV, respectively, was considered the optimum formulation. It was characterized by differential scanning calorimetry, examined using a transmission electron microscope, and a stability study was conducted at different temperatures to monitor its stability efficiency showing that NAR-HNP formulation maintains stability at 4 °C. The selected formulation was subjected to an acute toxicological test, a pharmacokinetic analysis, and a Diabetes mellitus (DM) experimental model. STZ (50 mg/kg) given as a single i.p. rendered rats diabetic. Diabetic rat groups were allocated into 4 groups: one group received no treatment, while the remaining three received oral doses of unloaded HNPs, NAR (50 mg/kg), NAR-HNPs (50 mg/kg) and NAR (50 mg/kg) + peroxisome proliferator-activated receptor-γ (PPAR*-*γ) antagonist, GW9662 (1mg/kg, i.p.) for three weeks. Additional four non-diabetic rat groups received: distilled water (normal), free NAR, and NAR-HNPs, respectively for three weeks. NAR and NAR-HNPs reduced immobility time in forced swimming test and serum blood glucose while increasing serum insulin level. They also reduced cortical and hippocampal 5-hydroxyindoeacetic acid, 3,4-Dihydroxy-phenylacetic acid, malondialdehyde, NLR family pyrin domain containing-3 (NLRP3) and interleukin-1beta content while raised serotonin, nor-epinephrine, dopamine and glutathione level. PPAR*-*γ gene expression was elevated too. So, NAR and NAR-HNPs reduced DM-induced depression by influencing brain neurotransmitters and exhibiting anti-oxidant and anti-inflammatory effects through the activation PPAR-γ/ NLRP3 pathway. NAR-HNPs showed the best pharmacokinetic and therapeutic results.

## Introduction

Diabetes mellitus (DM) is a hormonal and metabolic condition distinguished by insulin resistance or a partial/entire deficiency of pancreatic insulin production, causing persistently elevated levels of glucose in the blood^1^. DM prevalence continues to rise globally, reaffirming diabetes as a major global threat to the health and well-being of individuals, families, and societies. Besides, DM is counted as one of the global health crises progressing at one of the quickest rates in the twenty-first century^2^. Based on the International Diabetes Federation (IDF) report, it is anticipated that there will be 643 million individuals living with diabetes by 2030, and 783 million by 2045^3^. Notably, DM is frequently associated with severe complications, such as, diabetic kidney damage, depression, diabetic foot ulcer, diabetic retinal damage, and peripheral arterial disease^4^.

Diabetes and depression co-occurring are detrimental to both lifestyle and quality of life^5^. Depression is threefold more common in patients with type 1 DM and twofold in those with type 2 DM^6^. Even though significant rates of depression are seen in people with DM, the pathophysiology of co-morbid depression and diabetes is still not completely understood^7^. DM and depression share multiple routes in their pathophysiology, including immune-inflammatory mediators, endocrinological variables, and neurological components^5^. STZ is a naturally occurring alkylating chemical that is especially harmful to pancreatic β-cells, it is often used to induce experimental type 1 DM in rats. Experimentally, diabetic rats induced with STZ exhibited depressive-like behavior when subjected to the forced swimming test (FST), a well-established test in the field of depression^8^.

Chronic hyperglycemia has been shown to enhance free radical buildup, which leads to inflammation and reactions related to inflammation^9^. Studies have shown that depression and diabetes are linked to high levels of inflammasomes, particularly NLR Family Pyrin Domain Containing Protein 3 (NLRP3)^10,11^.

PPARs, also known as peroxisome proliferator-activated receptors, are nuclear hormone-activated receptors that come in three distinct subtypes: PPAR-γ, PPAR-α, and PPAR-β/δ^12^. Many biological processes, including insulin sensitivity, metabolism of fatty acids, immunological responses, in addition to cell development and differentiation, are thought to be significantly regulated by PPARs^13^. It is worth noting that DM is accompanied by downregulation of the expression of PPARγ^14^. PPARγ possesses an anti-oxidant effect and has been linked to a reduction in depression symptoms^15^. PPARγ-receptors are expressed in certain parts of the brain related to depression, including the hippocampus, basal ganglia, frontal cortex, hypothalamus, and pituitary. It has been shown that activating these receptors helps a variety of CNS dysfunctions, including depression^16^. Multiple in vitro and in vivo experiments have demonstrated that PPARγ suppresses nuclear factor-kappa β (NF-kβ) nuclear transcription factor, thus preventing the evolution of pro-inflammatory cytokines and oxidative stress. In this sense, PPARγ may be a good therapeutic target for discovering new drugs to treat mood disorders^17^.

Naringenin (NAR) is a flavanone found naturally in citrus fruits, cherries, and tomatoes^18^. Numerous pharmacological activities of NAR have been described, including anti-inflammatory, anti-diabetic, anti-fibrotic, anti-obesity, and anti-dyslipidemic activity^19^. In addition, NAR has the potential to chelate metals and also acts as an anti-oxidant by neutralizing free radicals^20^. It has been reported that NAR enhances insulin sensitivity and secretion, reduces glucose production, combats oxidative stress, and inflammation in diabetes^21^. Besides, NAR was shown to have a marked modulatory influence on PPARγ receptor expression and the translocation of muscle glucose transporter 4 (GLUT-4)^22^. Despite the various pharmacological actions of NAR, its poor oral bioavailability limits its practical use. The poor bioavailability was attributed to low aqueous solubility, diminished gastric absorption and extensive metabolism by cytochrome-P450 as well. NAR administrated orally to humans and animals showed less than 10% bioavailability. Accordingly, the development of a NAR formulation with a better bioavailability is necessary^23–25^.

Nanoparticulate drug delivery systems have been developed as promising carriers for enhancing the solubility of sparingly soluble drugs, raising their bioavailability, and providing sustained release^26,27^. Lipid polymer HNPs are advantageous as a drug delivery platform because they have superior in vitro and in vivo stability, controlled particle size, sustained drug release profile, and high drug loading yield^28,29^.

In this study, Chitosan (CS) was chosen as the polymer because it is one of the interesting natural polymers that has shown effective results in drug delivery^30,31^. In addition to being a non-toxic polymer, biodegradable, and possessing anti-microbial activity^32^. Glyceryl monooleate (GMO) was chosen as the lipid. The biocompatible food additive GMO has long, unsaturated hydrophobic chains^33^. Its presence increases CS hydrophobicity and thus enhance binding affinity between the carrier and the drug. GMO and CS is considered as a distinctive nanoparticulate system possessing capability to keep the encapsulated drug and increase its stability as well as achieving drug delivery to the target sites^34^.

Though it was reported that NAR modulates oxido-inflammatory insults and brain neurotrophic factors expressions to alleviate depressive behavior in mice exposed to hypoxic stress^35^, it restored changes in the kynurenine pathway, demonstrating a strong neuroprotective effect in olfactory bulbectomized-mice model of depression^36^, and has shown an anti-depressant effect in a rat model of chronic unpredictable mild stress^37^, it’s anti-depressant effect on STZ-induced diabetes in rats has not been demonstrated yet. To the best of our knowledge, no research on the synthesis of NAR-loaded chitosan/GMO nanoparticles possessing an anti-depressant effect on STZ-induced diabetes in rats has been investigated yet. Thus, the current study assesses the validity of NAR and NAR-loaded hybridized nanoparticles (NAR-HNPs) as an oral drug delivery system, for NAR in an attempt to enhance its oral bioavailability in STZ-induced depressed rats. Moreover, glycemic control, oxidative stress biomarkers, PPARγ/NLRP3 pathway, and brain monoamines were assessed.

## Materials and methods

### Drugs and chemicals

Sigma Chemical Company in St. Louis, Missouri, provided streptozotocin (STZ), Naringenin (NAR), the low M. Wt chitosan (50,000–190,000 Da), GMO, Poloxamer 407 (P407), and cellulose membrane (molecular weight cutoff 12,000–14,000 Da). Lipoid GmbH, Switzerland, kindly donated Lipoid S45. El-Nasr Company for Pharmaceutical Chemicals, Cairo, Egypt, provided all other reagents, which were all of analytical grade.

### Preparation of hybridized nanoparticles

The previously reported self-assembly method^38–40^ was used for the preparation of NAR-HNPs with modifications. Briefly, 50 mg NAR dispersed in aqueous solution of 0.25% w/v poloxamer F12750 mg was added dropwise under magnetic stirring to 1% v/v aqueous acetic acid solution containing CS (0.2 or 0.4% w/v). The lipid matrix was prepared as 2% w/v or 4% w/v GMO with or without 2% w/v lipoid S45 dissolved in 5 ml ethanol. The drug polymer solution was gradually supplemented with the lipid matrix. The resultant mixture was magnetically stirred for 30 min at room temperature. After that, the mixture was placed in a water bath at room temperature and subjected to a probe-type ultrasonicator treatment for 5 min at a power level of 150W to create NAR-loaded lipid polymer nanoparticles. A rotary evaporator (Rotavapor, Buchi-M/HB-140, Switzerland) evaporated the organic solvent at 45 °C while operating under vacuum. The resulting NAR-HNPs were stored in refrigerator (4 °C)^41^, for further study after being allowed to cool to ambient temperature.

### Characterization of NAR-loaded hybridized nanoparticles

#### Entrapment efficiency measurement (EE%)

The entrapment efficiencies of the formulated nanoparticles were calculated indirectly through the separation of the unentrapped NAR from the one that was trapped in the NAR-HNPs by centrifugation at 10,000 rpm for 60 min at 4° (Union 32R, Hanil, Korea). The supernatant was collected, passed through a 0.2-micron Millipore membrane filter, and then suitably diluted with methanol before being spectrophotometrically analyzed. at 289 nm (Shimadzu UV spectrophotometer, 2401/PC, Japan)^42,43^. The entrapment efficiency was calculated using the following equation:

$$ {\text{EE}}\% \, = \frac{{{\text{Total NAR}} - {\text{Free NAR}}}}{{\text{Total NAR}}} \times 100 $$

### Particle size analysis (PS)

The particle size measurement was conducted after tenfold appropriate dilution of the NAR-HNPs with distilled water using a Zetasizer (Malvern Instrument, Worcestershire, UK) at a fixed angle of 90° at 25 °C. Each value was the average of three measurements.

### Zeta potential measurement

The particle charge was measured using a Zetasizer at 25 °C. Each sample was required to be sufficiently diluted tenfold with demineralized particle-free water before measurement.

### In vitro release of NAR from the hybridized nanoparticles

The dialysis bag diffusion technique was used to compare the in vitro release of NAR from NAR-HNPs to the free NAR^44^. The sample was put in the dialysis membrane, sealed at both ends, and then submerged in 100ml of release media (phosphate buffer pH 6.8) that was stirred at 50 rpm and kept at 37 ± 0.5 °C^45^. 2 ml of the sample was taken out of the release medium and replaced with the same volume of the fresh release medium at the predetermined time intervals (0.5, 1, 2, 3, 4, 5, 6, 7, 8 and 24 h). At 322.8 nm, the amount of NAR released was quantified spectrophotometrically. The experiment was done in triplicate, and the results were presented as mean values with standard deviations. A graph showing the cumulative percentage of NAR released over time was illustrated.

### Characterization of the selected NAR-HNPs

Regarding the results obtained from the entrapment efficiency (EE), particle size (PS) analysis, zeta potential (ZP) measurement and the in vitro release of NAR from the prepared HNPs, the formulation showing the highest EE%, acceptable PS and maximum percent of NAR released after 24 h was chosen for further characterization and for the in vivo evaluation.

### Differential scanning calorimetry (DSC)

The thermal characteristics of NAR, CS, lipoid S45, and the optimized NAR-HNPs were determined using DSC (Shimadzu DSC- 60 apparatus, Shimadzu Corporation, Kyoto). About 5 mg sample was weighed accurately into standard aluminum pans using an empty pan as a reference. Under liquid nitrogen (25 ml/min), the samples were heated from 30 to 300 °C at 10 °C per minute. The thermograms generated were documented and the effects of NAR inclusion in the nanoparticles were assessed.

### Morphology examination of NAR-HNPs

Transmission electron microscopy (JEOL, JEM-1230, Tokyo, Japan) was used to examine the morphology of the optimized NAR-HNPs. One drop of the diluted sample was stained with 2% (w/v) phosphotungestic acid for 30 seconds at room temperature, and then it was deposited on carbon-coated grids with films for analysis.

### pH measurement

The pH of selected formulation was determined at a temperature of 25 °C using a pH meter (Jenway, Bibby Scientific Limited, Staffordshire, UK). The pH meter was originally standardized using buffer solutions with pH values of 7.0 and 10.0. 0.5 gm of NAR-HNPs had been completely mixed and diluted ten times with bi-distilled water, pH was determined. Three measurements, including the mean and standard deviation, produced the values that were displayed.

### Study of NAR-loaded HNPs stability

The impact of one month storage on both physical and chemical properties was examined^46,47^, to test the stability of the optimized formulation. NAR-HNPs was kept in screw-capped vials in the dark and stored at 4 °C, 25 °C, and 40 °C^48^. Every 10 days, samples were taken, evaluated for EE%, PS, ZP, and PDI, and then compared to freshly made formulation. Three duplicates of each experiment were carried out.

### Acute toxicity study in rats

Male and female adult Wistar rats that weighed between 200 and 230 g were acquired from the Animal House Colony of the National Research Centre in Giza, Egypt. The study was done based on prior research^49^. On the treatment day, rats were distributed arbitrarily to one of three groups, each containing ten rats (five females and five males), with group 1 served as the normal, group 2 received unloaded HNPs, and finally, group 3 received NAR-HNPs. Each animal was observed for signs of toxicity, daily for 2 weeks. Rats were visually inspected to detect skin, membrane, and respiratory changes. Trembling, tremors, saliva production, diarrhea, tiredness, sleep and coma were all noticed.

### Hematological analysis

White blood cells (WBCs), hematocrit (HCT), Red blood cells (RBCs), hemoglobin concentration (Hb), and platelet count (PLT) were analyzed from blood samples using an automated cell counter, the Medonic M20 cell counter (Boule Medical AB, Stockholm, Sweden which is present in the Medical Research Center of Excellence in the National Research Centre, Egypt).

### High-performance liquid chromatography verification method

HPLC (High-Performance Liquid Chromatography) method verification involves confirming that a specific HPLC analytical method is suitable for its intended purpose and meets predefined acceptance criteria. This process was performed according to ICH guidelines and ensures that the method is reliable, accurate, and provides consistent and precise results.

### Linearity

The acceptable range for linearity in analytical chemistry, particularly for calibration curves in methods like HPLC, The R2 value should generally be close to 1.0 (preferably greater than 0.99). It represents the proportion of the variance in the dependent variable (response) that is predictable from the independent variable (concentration).

### Precision and accuracy

The International Council for Harmonization of Technical Requirements for Pharmaceuticals for Human Use (ICH) provides guidelines for the pharmaceutical industry, including guidance on validation of analytical procedures. Acceptance criteria for precision are typically defined in terms of relative standard deviation (RSD) or percentage relative standard deviation (%RSD) for peak area. However, specific %RSD values ranged between 2 and 5%.

In addition, in HPLC, accuracy is a fundamental parameter that ensures the measured results from the method are close to the true or accepted values for the analyzed analytes. The accuracy limit in HPLC is typically expressed as a percentage relative error or percentage recovery. It's important to achieve accurate results to ensure the reliability and validity of the analytical data obtained. The acceptable accuracy limit in HPLC is often defined within a specific range, commonly between 98 and 102%.

### Pharmacokinetic study of NAR

Rats (200 and 230 g) were randomly divided into 2 groups, 6 rats each. Rats were food-fasted for 12 h. Rats in group 1 were orally administered free NAR (50 mg/kg) suspended in sodium carboxymethylcellulose (0.5%). Group 2 rats were orally administered an equivalent dose of NAR-HNPs (50 mg NAR dispersed in aqueous solution of 0.25% w/v poloxamer F12750 mg was added dropwise under magnetic stirring to 1% v/v aqueous acetic acid solution containing CS (0.2 or 0.4% w/v). Following drug administration, blood samples (about 0.5 ml) were drawn at 0.25, 0.5, 1, 2, 3, 4, 6, 8, 24, and 48 h. Blood samples were centrifuged right away for 10 min; separated plasma were aliquoted and kept at − 80ºC until analysis. Dose of NAR was chosen in relevance to previous study^50^.

### Preparation of plasma samples

Samples were extracted twice with ethyl acetate, stirred for three minutes, and centrifuged for five minutes at 3,500 rpm. The combined supernatants were then dried and reconstituted with acetonitrile, and 20 μl of the reconstituted supernatant was injected into HPLC for quantitation.

### Instrumentation and chromatographic conditions

The Agilent HPLC system (HP 1100 series), which includes a quaternary pump, on-line degasser, auto-sampler, and UV detector, was used to analyse the plasma samples under the control of the Chemstation software (Hewlett Packard, Waldbronn, Germany). Agilent Zorbax XDB C18 column (150 × 4.6 mm I.D., 5 m, Germany) served as the analytical column, and a guard cartridge containing a comparable stationary phase served as the guard. NAR was isocratically eluted at pH 2.5 and 1.0 ml/min using acetonitrile, water, and formic acid (21:78.8:0.2). The 280 nm detection wavelength. By comparing the peak areas of the sample to those of standard solutions of NAR, the NAR concentrations in the plasma samples were calculated^51^.

### Pharmacokinetics and statistical analysis

The pharmacokinetic parameters for NAR or NAR-HNPs were derived using the PK Solver 2.0 'add-in' for Excel 2010 software. The data were presented as mean ± SEM. GraphPad Prism 8.0 for Windows was employed for all statistical analyses (GraphPad, San Diego, CA, USA).

### Induction of diabetes

Before use, 0.1 M citrate buffer (pH 4.5) was used to liquify 50 mg/kg of streptozotocin (STZ)^52^. 26.75 ml of citric acid solution (1.05 g of citric acid in 50 ml of distilled water) and 21.75 ml of sodium citrate solution (1.05 g of sodium citrate in 50 ml of distilled water) were mixed to prepare the buffer, and the volume was increased to 100 ml of distilled water. To lower hypoglycemia shock-related mortality, a 5 percent glucose solution was given to all rats for 24 h after STZ administration instead of tap water. Blood samples were collected from rats’ tail veins two days after STZ treatment, and blood glucose levels were subsequently measured using a portable glucometer (ACCU-Check, Roche, USA). Rats with 200 mg/dl or higher blood glucose levels were regarded as diabetic^53,54^.

### Experimental design

**Figure 1:**
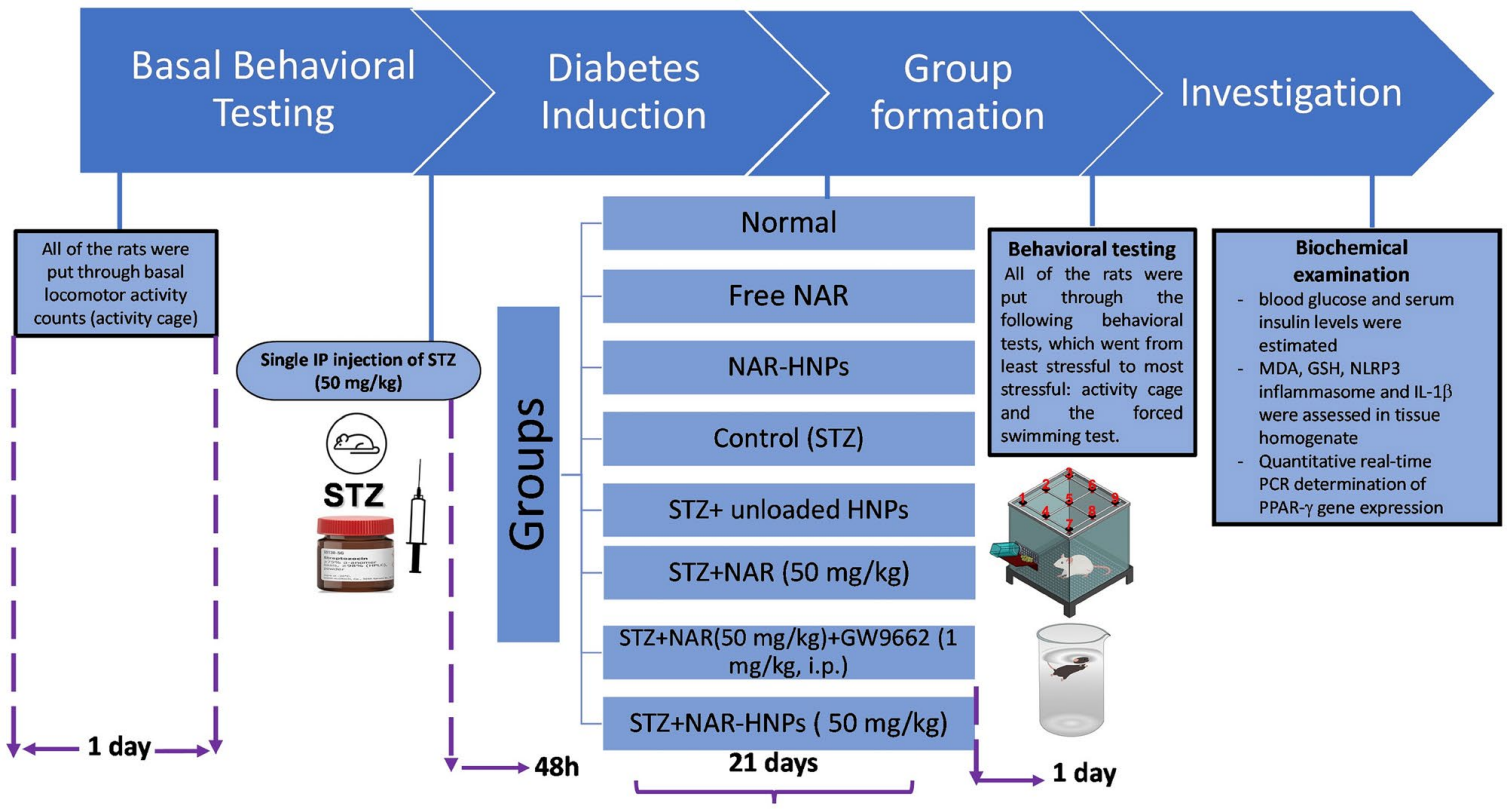
Schematic summary of the experimental study.

In the present study, 64 rats were randomly assigned into eight groups (8 rats per group) as demonstrated in Fig. 1. In group I, rats received distilled water for 21 days, and this group was considered as normal. In groups (II-III), rats received free NAR (50 mg/kg, p.o.) and NAR-HNPs (50 mg/kg, p.o.), respectively for 21 days. Group IV, considered as a control group and included STZ-induced diabetic rats (STZ group). Group (V-VIII), diabetic rats received unloaded HNPs, NAR (50 mg/kg, p.o.), NAR-HNPs (50 mg/kg, p.o.), and NAR (50 mg/kg, p.o.) + GW9662 (1 mg/kg, i.p.), respectively for 21 days. GW9662 was administered 15 min before NAR administration and it’s dose was chosen in accordance to prior study^55^.

### Body weight change

On both the initial and final days of the experiment, each animal's body weight was measured, and the following formula was used to figure out the percentage change in body weight:

$$ \% \;{\text{Change}}\;{\text{in}}\;{\text{body}}\,{\text{weight}} = \frac{{{\text{Body}}\;{\text{weight}}\;{\text{in}} \;{\text{first}}\;{\text{day - body}}\;{\text{weight}}\;{\text{in}}\;{\text{last}}\;{\text{day}}}}{{{\text{Body}}\,{\text{weight}}\;{\text{in}}\;{\text{first}}\;{\text{day}}}} \times 100 $$

### Behavioral analysis

#### Activity cage test

A grid floor activity cage (Model No. 7430, Ugo-Basile, Comerio, Italy) was employed to assess locomotor activity. The rat's movements that disrupted infrared beams were automatically tracked, and the apparatus software used this information to count the number of times the rat moved horizontally. Before administering STZ, rats were acclimatized to the test room for 1 h. Then, each rat was put in the activity cage for 5 min, and the basal activity counts were written down. When the test is over, each rat was removed carefully from the apparatus and returned to its home cage. To prevent odor cues, the arena was cleaned with a 70 percent (v/v) alcohol solution in distilled water after each rat. Each rat was tested again for 5 min with the activity apparatus 24 h after the drug was given, and the final activity counts were written down^56^. The formula used to determine basal activity counts as a percentage was:

$$ \% \;{\text{of}}\;{\text{basal}}\;{\text{activity}}\;{\text{counts}} = \left( {{\text{Final}}\;{\text{activty}}\;{\text{counts}}/{\text{Basal}}\;{\text{activity}}\,{\text{counts}}} \right){ } \times { }100 $$

### Forced swimming test

A vertical cylinder made of plexiglass measuring 46 cm in height and 20 cm in width was filled with tap water heated to between 23 and 25 °C to a depth of 30 cm. After 23 days of STZ injection, the FST was performed to the animals. Each rat was placed in the Plexiglas cylinder containing water for two swimming sessions. During the pre-test, the rat was required to swim for 10 min. Following the pre-test, the rat was withdrawn from the cylinder, dried, and returned to its home cage. After each test, the water in the cylinder was refilled. The rat was re-subjected to the same experimental settings for 5 min 24 h later. A camera above the cylinder filmed the test so that it could be studied later. The rat was immobile when it ceased moving and floated in the water with its head just above the surface, without moving^57^.

### Blood glucose level

A portable glucometer (OKmeter, OK Biotech) was used to assess blood glucose levels 24 h after the last treatment. A drop of blood was taken from each rat using the tail vein puncture technique and placed on the glucometer strip loaded in the device for blood glucose measurement.

### Sample preparation

Rats were decapitated after behavioral testing. Cortical and hippocampal brain tissues were extracted, immediately weighed to prevent any effects from drying, and stored at 80 °C after a meticulous brain removal. Other brain samples were used for quantitative real-time PCR analysis.

The homogenizer MPW-120 from medical instruments homogenized cortical and hippocampal brain tissues in 10% (w/v) ice-cold phosphate buffer. The homogenate was centrifuged using a cooling centrifuge (2k15; Sigma/Laborzentrifugen) at 4000 rpm for 5–20 min. The collected supernatant was then used to measure biochemical analysis.

### Enzyme-linked immunosorbent assay

In line with the manufacturer's guidelines, rat-specific enzyme-linked immunosorbent assay (ELISA) kits (Catalog No: SL0475Ra, SL0402Ra, SL1158Ra, SL1497Ra, SL0373Ra and SL0998Ra SunLong Biotechnology Co., LTD, HangZhou, China) were used to assess malondialdehyde (MDA), interleukin-1β (IL-1β), NLRP3, insulin, and reduced glutathione (GSH) in cortical and hippocampal homogenate.

### Gene expression of PPARγ

According to the manufacturer’s guidelines, total RNA was isolated from brain (hippocampus and cortex) tissue with *GENEzol*™ TriRNA Pure kit (Geneaid®). The level and purity of RNA were determined with Nanodrop spectrophotometer. RevertAid-first sreand cDNA synthesis kit (Thermo Fisher®invitrogen ™) was used to synthesize cDNA from total RNA(2.0µg) according to manufacturer’s guidelines.

Rotor-Gene Q MDX instrument was used to perform the real-time PCR amplification. Each reaction of RT-PCR (20µl) was consisted of cDNA (1.5µl), primer pairs (0.25µM), EverGreen master mix (4µ) (HOT FIREPol® EvaGreen® qPCR Mix Plus, Solis BioDyne™), and PCR water. Primer pairs sequence was adapted from literate. Proliferator-activated receptor (PPAR-γ) primer pair sequence^58^ was as follow: For-ttatagctgtcattattctcagtgga; Rev-cgggtggttcagcttcag. Glyceraldehyde 3-phosphate dehydrogenase primer pair sequence^59^ was as follows: For-gtattgggcgcctggtcacc; Rev-cgctcctggaagatggtgatgg. PCR amplification condition started at 50 °C for 2 min then 95 °C for 12 min followed by 45 cycles of 95 °C (20 Sec), 60 °C (30 Sec), 72 °C (30 Sec). The final step was the melting curve programme from 60 °C to 95 °C. PPAR-γ relative expression was calculated using delta-delta CT method^60^ and normalized to the house-keeping gene expression (GAPDH).

### Determination of monoamines content

The concentrations of cortical and hippocampal monoamines (NE, 5-HT, DA) as well as 5-Hydroxyindoleacetic acid (5-HTAA), and 3,4-Dihydroxyphenylacetic acid (DOPAC), metabolites in micrograms per gram of brain tissue were determined using HPLC (Agilent 1200 series; Agilent Technologies, California, USA) as previously described before^61^, and were calculated as follows: Where AT is the area under the curve for the sample, AS is the area under the curve for the standard, CS is the standard concentration (μg/ml), and the dilution factor is 10.

### Statistical analysis

Results are displayed as mean and SEM. Except for the activity cage test and stability study, in which two-way analysis of variance (ANOVA) followed by Tukey's multiple comparison test was used for analyses of activity cage test and two-way ANOVA followed by Bonferroni multiple comparison test was used for analyses of the stability study, all other analyses were conducted using one-way ANOVA followed by LSD test. Windows GraphPad Prism 8.0 was used for all studies (GraphPad, San Diego, CA, USA). The cutoff for statistical significance was *p* < 0.05.

### Ethics declarations

All methods were carried out in accordance with the relevant guidelines and regulations of the Medical Research ethics committee of the National Research Centre in Egypt. All experimental protocols were approved by the National Research Centre’s Medical Research ethics committee with approval number “19/217”. All methods are reported in accordance with ARRIVE guidelines.

## Results

### Preparation and characterization of NAR-HNPs

NAR-loaded HNPs were successfully prepared using the self-assembly method, and the detailed compositions are presented in Table 1. As evidenced by the nanoparticle’s composition, poloxamer F127, which has been proven to have a high affinity for GMO in creating liquid crystal nanoparticles, was utilized as a stabilizer to help incorporate GMO into the nanoparticles^39^. Moreover, the presence and absence of lipoid S45 during the NAR-HNPs preparation were studied. The results of the measurements for the EE%, PS, ZP, and polydispersity index (PDI) on the developed formulations are displayed in Table 2. Results clearly show that the increase in the concentration of both GMO and CS was accompanied by an increase of the EE in the range of 81.77 ± 2.10% to 99.3 ± 1.25%. Moreover, NAR-HNPs prepared with GMO in addition to lipoid S45 exhibited the highest EE-values, this may be possible due to higher concentration of the lipid^62^.

**Table 1:** Composition of the NAR-HNP formulations.

Formulae	CS (%w/v)	GMO (%w/v)	Lipoid S45 (%w/v)
NAR-HNP1	0.2	2	-
NAR-HNP2		4	
NAR-HNP3	0.4	2	
NAR-HNP4		4	
NAR-HNP5	0.2	2	2
NAR-HNP6		4	
NAR-HNP7	0.4	2	
NAR-HNP8		4

**Table 2:** Particle size, PDI, zeta potential, and entrapment efficiencies-values for the prepared NAR-HNPs.

Formulae	EE (% ± SD)	PS (nm ± SD)	ZP (mV ± SD)	PDI(± SD)
NAR-HNP1	81.77 ± 2.10	330.6 ± 8.45	33.9 ± 0.12	0.34 ± 0.01
NAR-HNP2	89.5 ± 2.40	348.0 ± 10.24	32.0 ± 1.02	0.31 ± 0.02
NAR-HNP3	91.22 ± 1.25	413.5 ± 12.01	48.0 ± 1.33	0.31 ± 0.01
NAR-HNP4	95.5 ± 2.00	419.6 ± 11.11	41.7 ± 0.25	0.46 ± 0.02
NAR-HNP5	96.8 ± 1.89	414.7 ± 12.35	51.1 ± 0.33	0.22 ± 0.03
NAR-HNP6	98.7 ± 2.01	415.2 ± 9.54	52.8 ± 1.04	0.21 ± 0.02
NAR-HNP7	97.8 ± 2.45	589.2 ± 8.25	53.4 ± 2.41	0.63 ± 0.05
NAR-HNP8	99.3 ± 1.25	643.1 ± 8.02	58.9 ± 2.95	0.82 ± 0.02

Moreover, NAR-HNPs prepared with Lipoid S45 exhibited the highest EE-values. Concerning the PS of the prepared NAR-HNPS, increasing the concentration of CS led to a remarkable (*p*<0.05) increase in nanoparticle size. All the developed formulations had particle sizes ranging from 330.6 ±8.45 nm to 643.1 ±8.02 nm. Simultaneously, results show that both PS and ZP are increased in HNPs prepared with Lipoid S45 than comparable HNPs prepared without Lipoid S45. As depicted from the results, the surface charge of the formulations prepared with or without lecithin remained positive, ranging from 32.0 ±1.02 mV to 58.9±2.95 mV. NAR-HNPs show low values for PDI ranging from 0.21±0.2 to 0.82±0.02.

### In vitro release study

**Figure 2:**
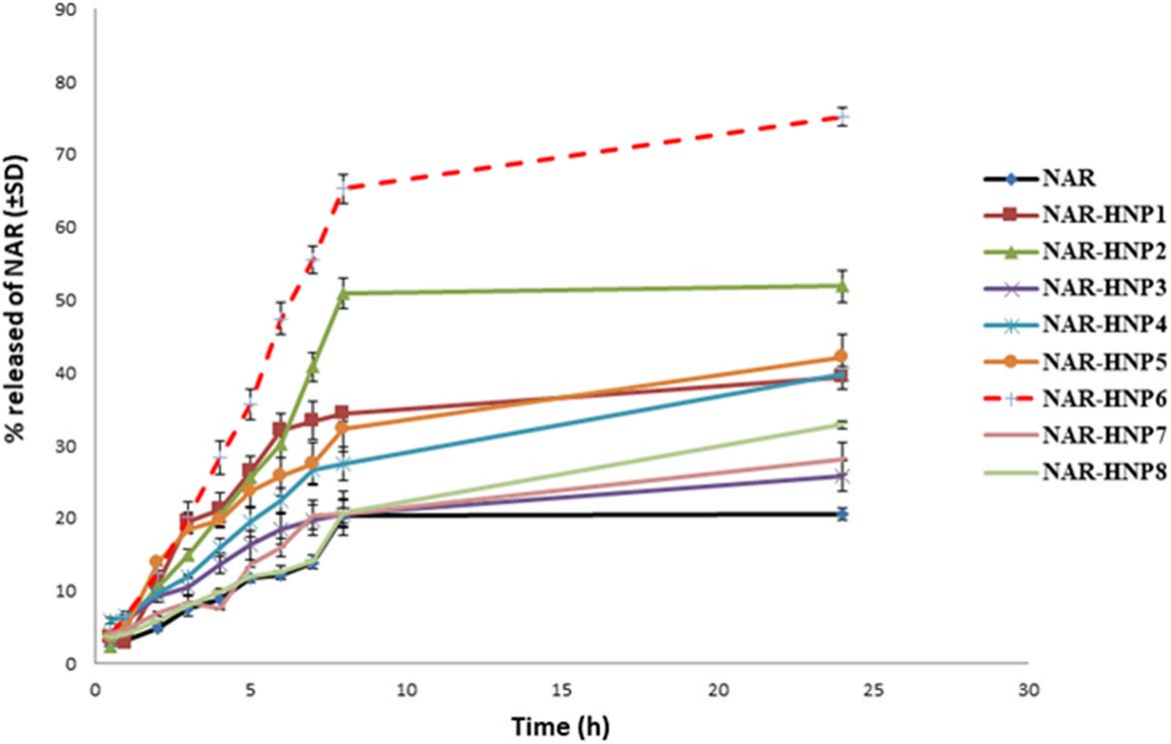
In vitro NAR release profiles of different hybridized nanoparticle formulations in comparison with the free drug in phosphate buffer saline pH 6.8 at 37 °C.

Figure 2 displays the release profile of various NAR-loaded NPs and free NAR. The results demonstrate that NAR has a slower release rate than the investigated NAR-HNPs. Comparing the studied formulations, NAR-HNP6 showed the highest percentage of NAR released up to 24 h. Furthermore, due to its high EE% and ZP, as well as its small PS and lowest PDI value, NAR-HNP6 was chosen as the optimum formulation for further investigation. The NAR-HNP6 DSC thermogram showed that neither NAR nor CS had any usual peaks.

### Characterization of selected NAR-HNP

#### Differential scanning calorimetry

**Figure 3:**
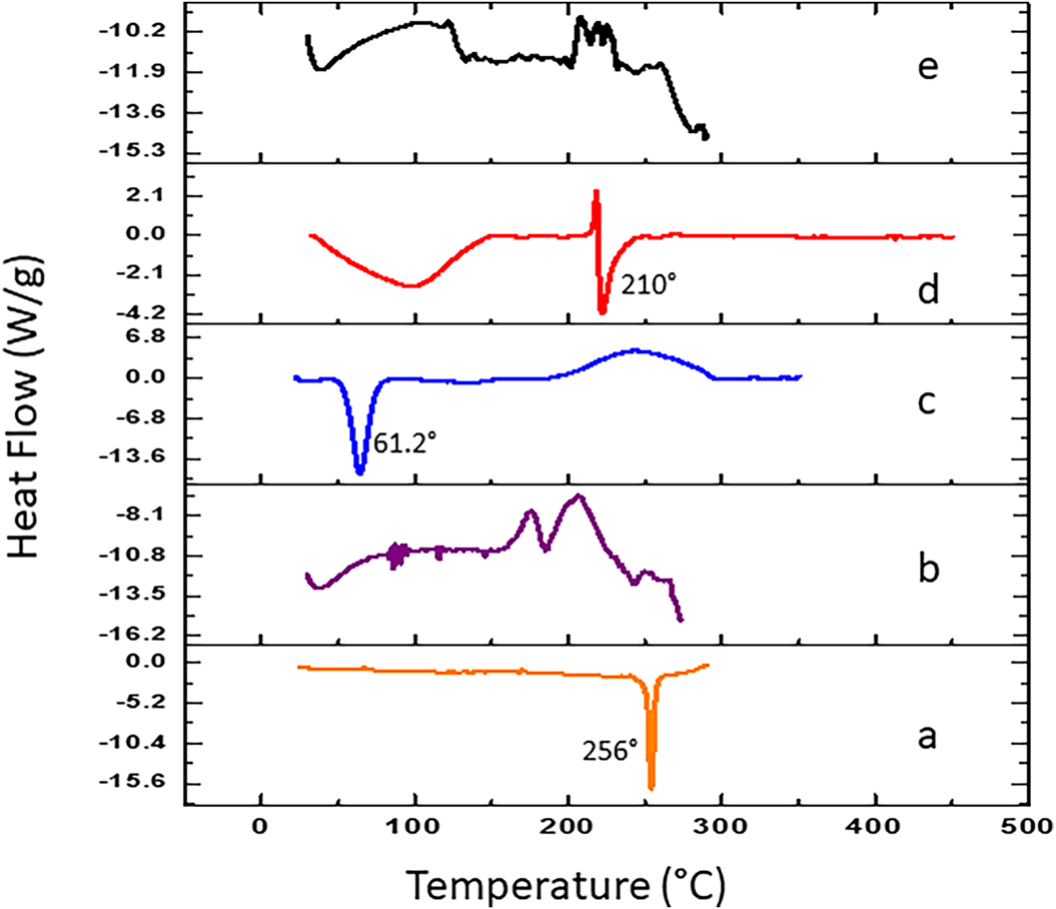
DSC thermograms of (**A**) NAR, (**B**) lipoid S45, (**C**) P407, (**D**) CS and (**E**) NAR-HNP6.

Figure 3 illustrates the DSC thermograms of NAR, lipoid S45, P407, CS, and NAR-HNP6. At 256 °C, NAR displays a pronounced endothermic peak, attributed to drug melting and indicating crystallinity^63^. The DSC thermograms of P407 and CS revealed endothermic peaks at 61.2 °C and 210 °C, corresponding to their melting temperatures. Lipoid S45's amorphous nature prevented it from exhibiting any identifiable peaks.

### Transmission *electron* microscopy

**Figure 4:**
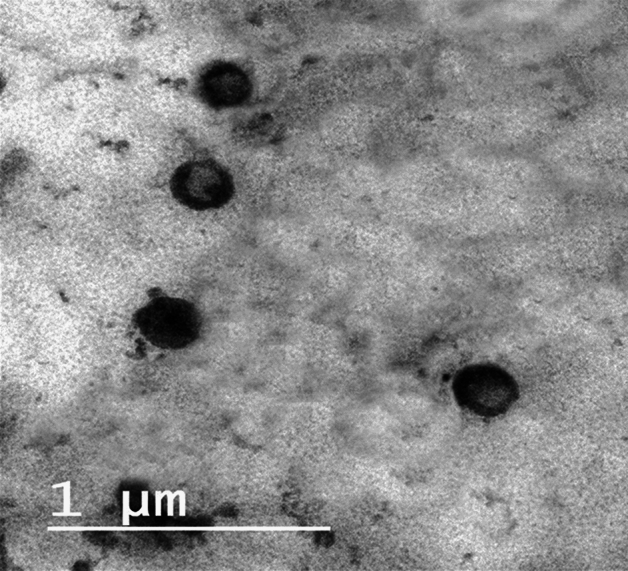
Transmission electron micrographs of NAR-HPN6.

Figure 4 depicts the micrograph of NAR-HNP6. The image shows well dispersed black dots, round, nanosized, and homogeneous nanoparticles with a compact structure, and a smooth surface.

### pH measurement

According to literature, a pH range of 2–9 can be accepted for oral solution^64^. Therefore, the pH of an oral drug must fall within this range. The pH value of NAR-HNP6 is 4.9 ± 0.02 which is suitable for oral administration.

### NAR-HNPs stability

**Figure 5:**
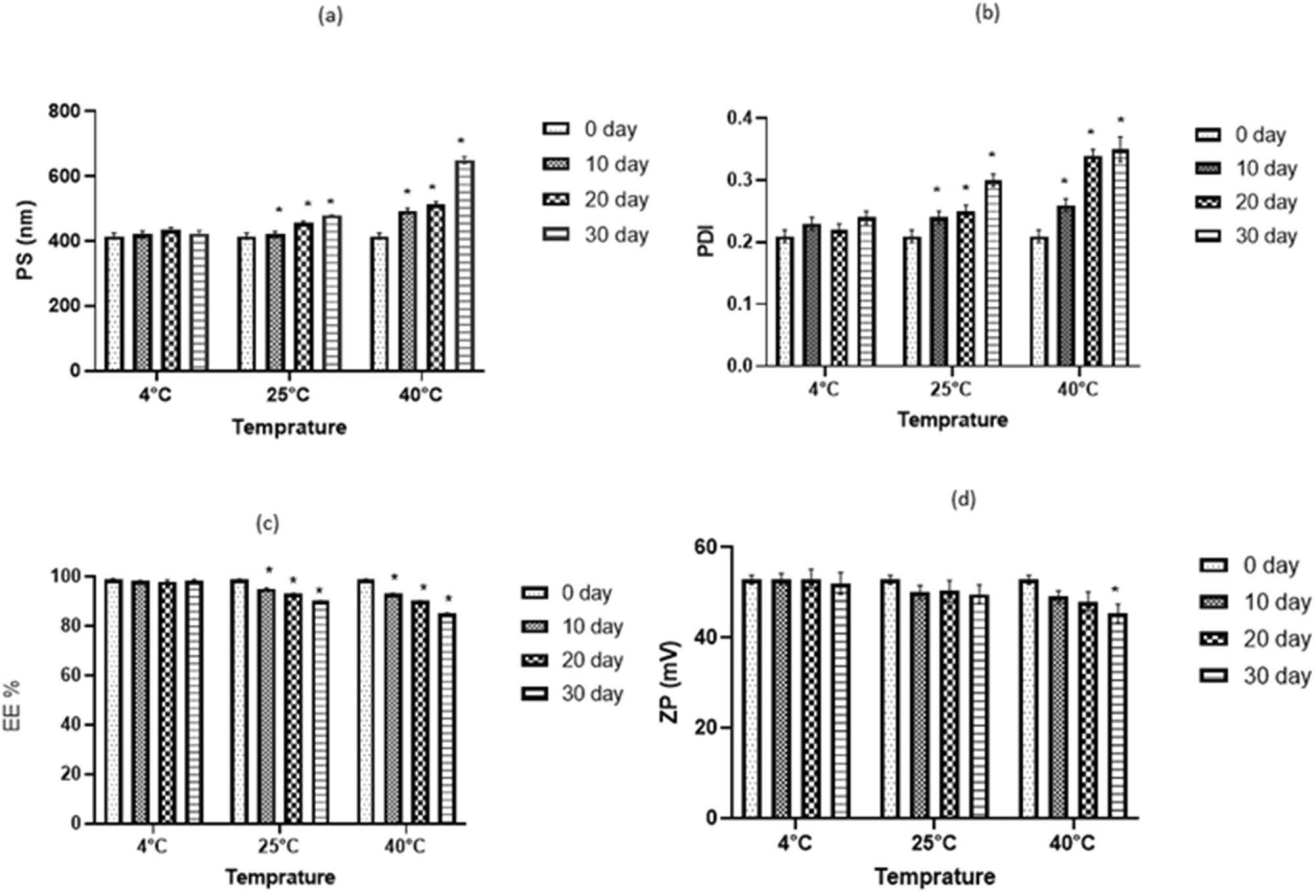
Storage effect on the particle size (**a**), polydispersity index (**b**), entrapment efficiency (**c**), and the zeta potential (**d**) of NAR-HNP6 at 4 °C, 25 °C and 40 °C. Results are stated as mean ± SD (n = 3). * vs correspondent o day.

Figure 5a–d depicts the impact of storage on EE%, PS, ZP, and PDI over 30 days at various temperatures, beginning on the first day of preparation. NAR-HNP stored at 4 °C did not show any indications of gelation, creaming, phase separation, or particle aggregation. However, an observed particle aggregation and creamy appearance were recorded at both temperature 25 °C and 40 °C. Additionally, NAR-HNP PS held at 4 °C did not change considerably (*p* = 0.9915) and displayed PDI values below 0.3 indicating a monodisperse pattern and a good homogenous population for the examined vesicles (*p* = 0.0973). On the other side, NAR-HNP stored at 25 °C and 40 °C exhibited an increase in PS and PDI accompanied with decrease in the entrapment efficiency (Fig. 5a–c). Additionally, as storage time was extended at 4 °C, the formulation absolute ZP value did not show significant change (*p* > 0.9999) (Fig. 5d), compared to that stored at 40 °C that demonstrated a significant decrease in the ZP values after 30 days of storage.

### Acute toxicity studies

**Figure 6:**
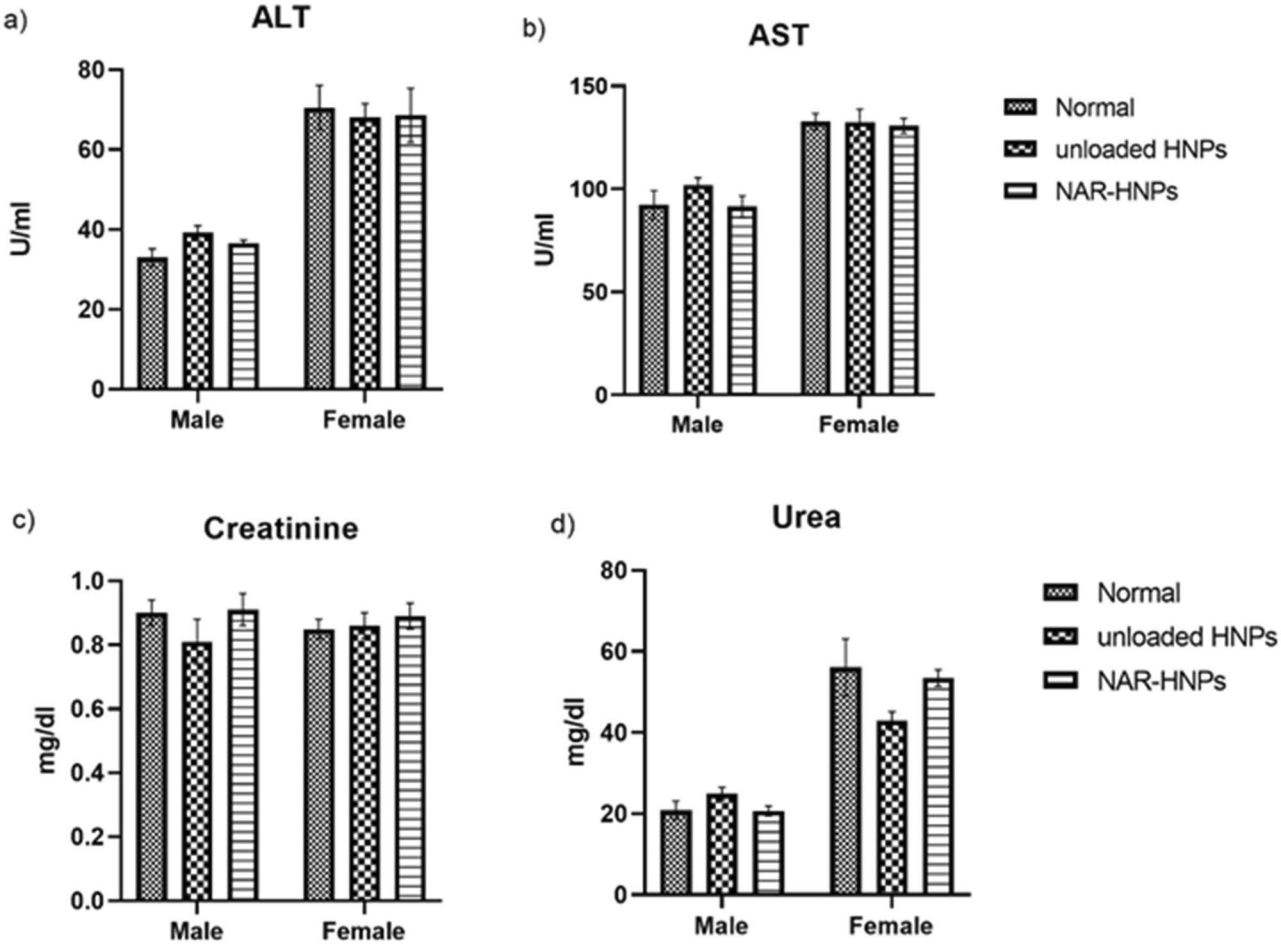
Effect of 2 weeks treatment of NAR and NAR-HNPs on (**a**) ALT, (**b**) AST, (**c**) Creatinine and (**d**) Urea in male and female rats. Results are expressed as mean ± SEM. HNPs, hybridized nanoparticles; NAR-HNPs, naringenin- loaded hybridized nanoparticles.

**Figure 7:**
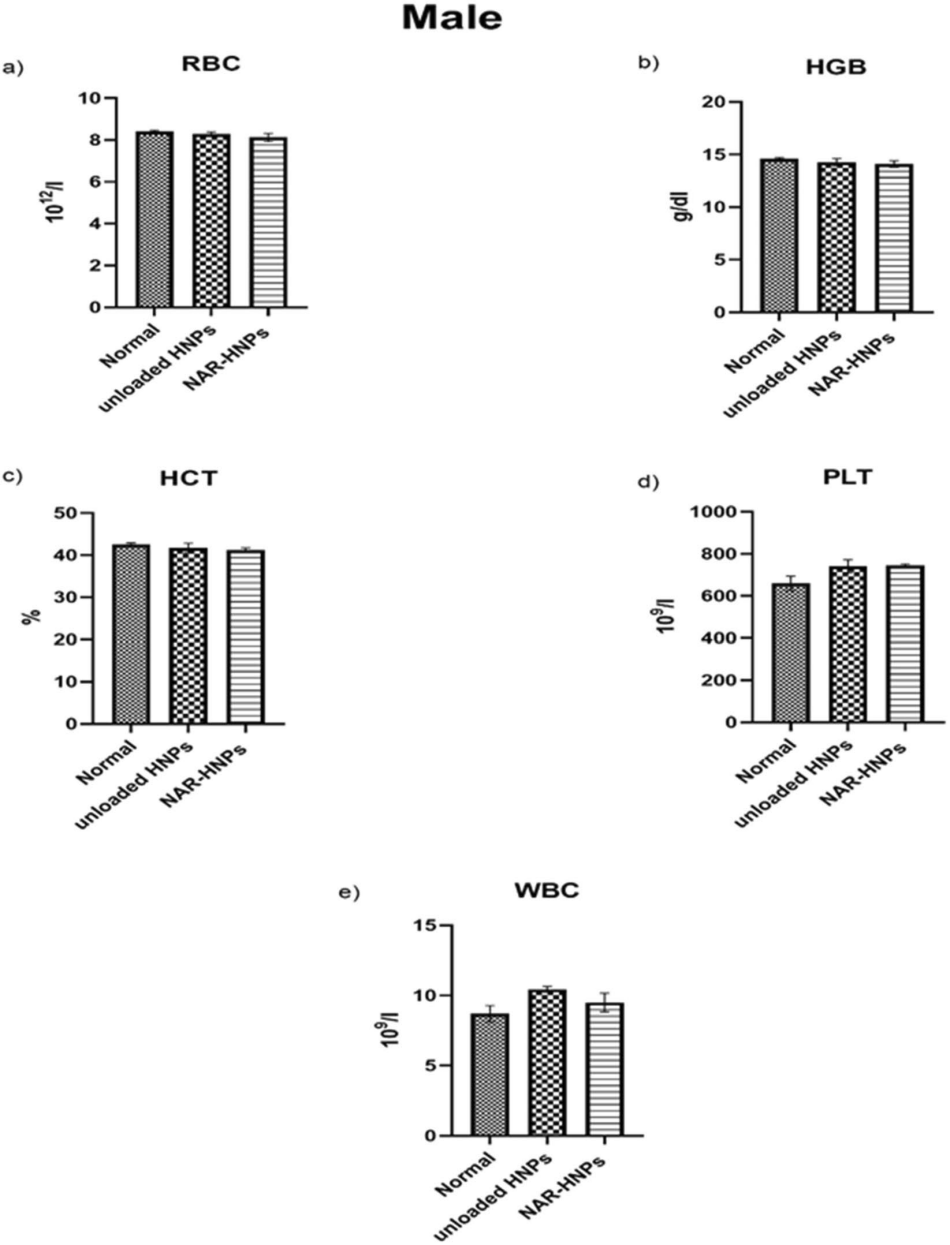
Effect of 2 weeks treatment of unloaded HNPs and the selected NAR-HNPs on (**a**) RBC, (**b**) HGB, (**c**) HCT and (**d**) PLT and (**e**) WBC in male rats. Results are stated as mean ± SEM. HNPs, hybridized nanoparticles; NAR-HNPs, naringenin- loaded hybridized nanoparticles.

**Figure 8:**
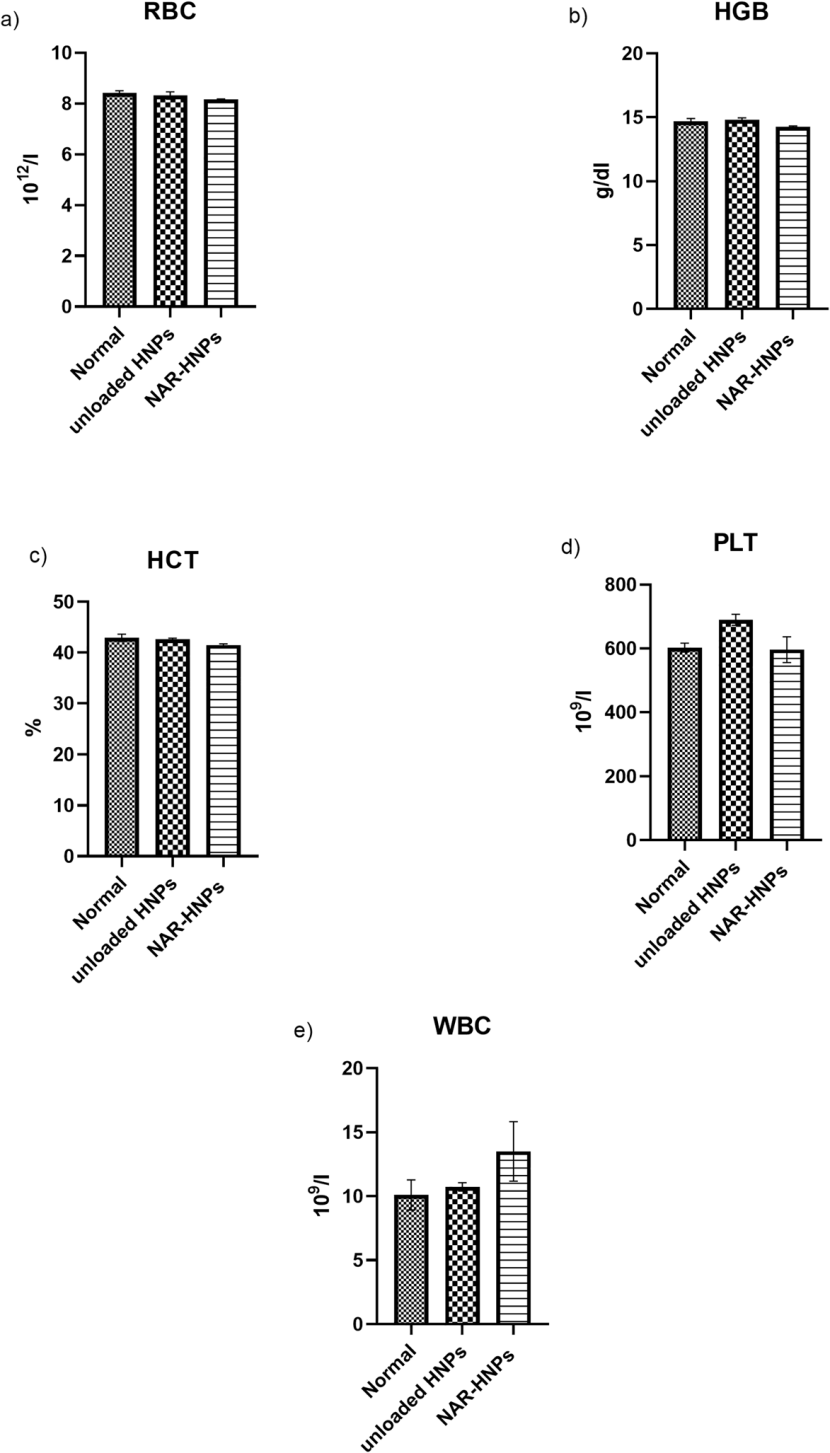
Effect of 2 weeks treatment of unloaded HNPs and the selected NAR-HNPs on (**a**) RBC, (**b**) HGB, (**c**) HCT and (**d**) PLT and (**e**) WBC in female rats. Results are stated as mean ± SEM. HNPs, hybridized nanoparticles; NAR-HNPs, naringenin-loaded hybridized nanoparticles.

As shown in Fig. 6, male and female rats administered NAR and NAR-HNPs exhibited no change in aspartate amino-transferase (AST) (F = 1.191; *p* = 0.3373); (F = 0.053;* p* = 0.9488), alanine amino-transferase (ALT) (F = 3.42; *p* = 0.0667); (F = 0.057; *p* = 0.9449) , urea (F = 1.93; *p* = 0.1877); (F = 2.47; *p* = 0.1261), and creatinine (F = 0.96; *p* = 0.4094); (F = 0.37; *p* = 0.7012), respectively. Concerning the hematological profile in both rats’ gender, no striking changes were depicted among the experimental groups as shown in Figs. 7, 8.

### Verification method in HPLC

**Figure 9:**
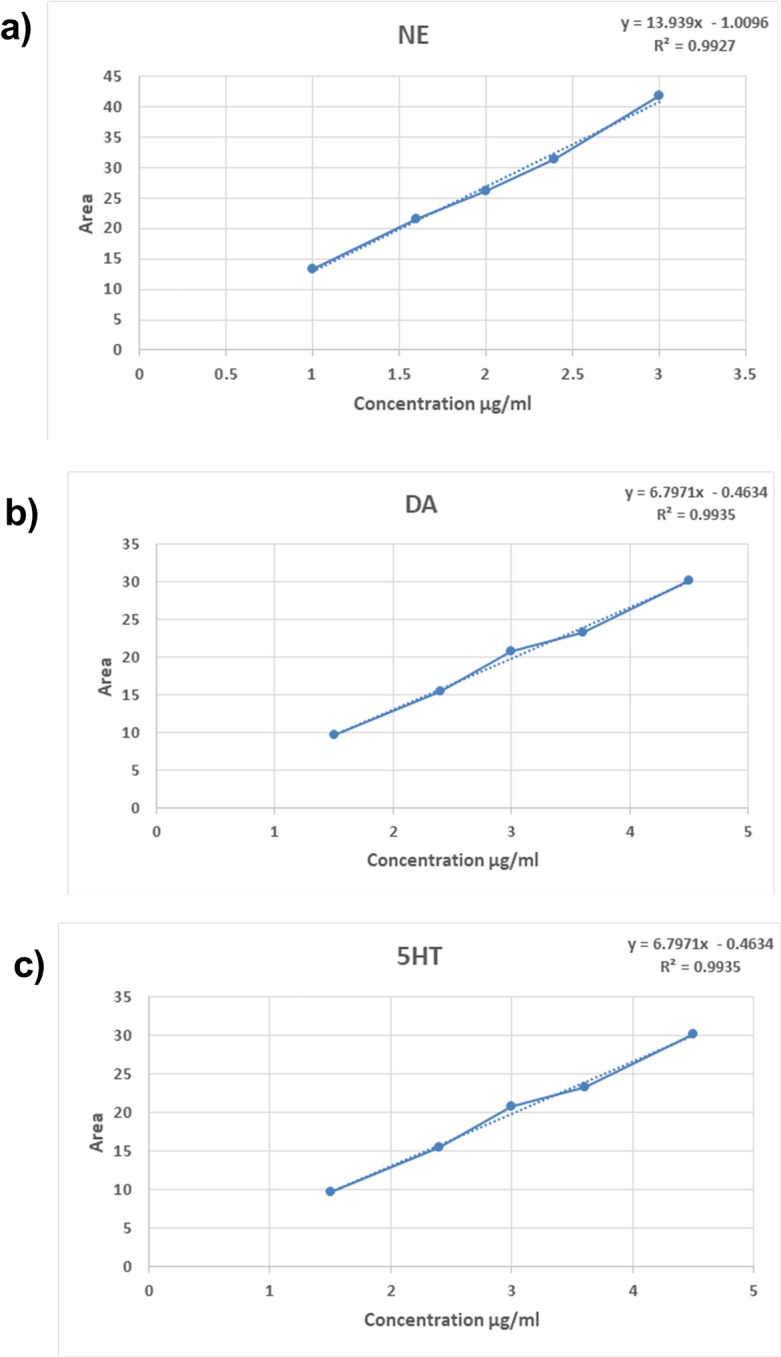
Linearity of NAR by HPLC verification method.

According to the presented data in Fig. 9, 10 the linearity of monoamines and NAR was in the optimum specification. Tables 3, 4, 5 depicts that the precision and accuracy of monoamines and NAR were in the optimum specification.

**Figure 10:**
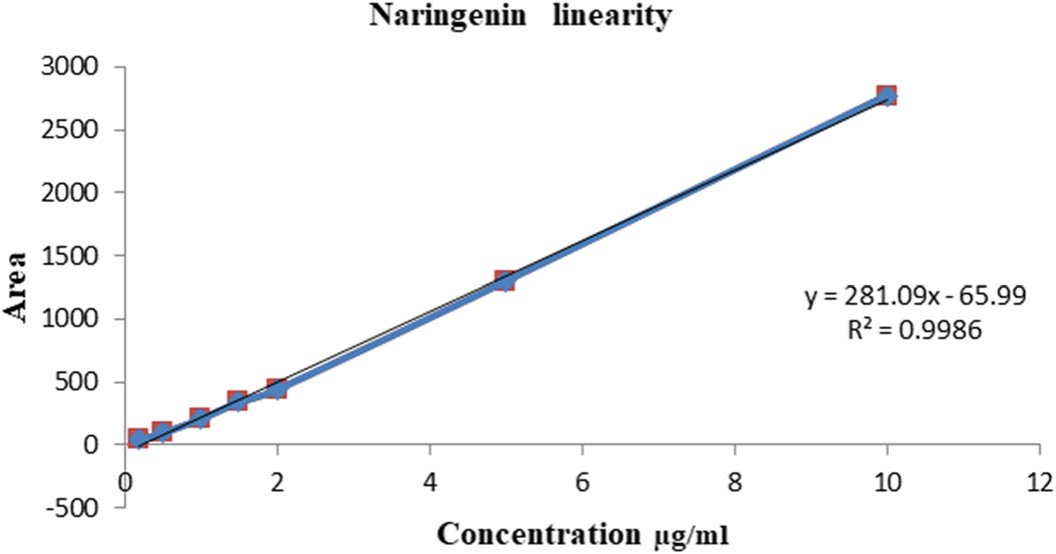
Linearity of Brain monoamines namely (**a**) NE, (**b**) 5-HT, and (**c**) DA by HPLC verification method.

**Table 3:** Precision results of monoamines in brain tissue.

Standard	Parameters	Precision (CV %)	
		Intraday	Interday
NE	LOD	0.115	0.114
	LOQ	0.345	0.350
DA	LOD	0.265	0.268
	LOQ	0.795	0.807
5HT	LOD	0.201	0.200
	LOQ	0.603	0.613

LOD: lower od detection; LOQ: Limit of quantification.

**Table 4:** Accuracy results of monoamines in brain tissue.

Accuracy			
Standard	% of linearity	Unfortified µg/ml	Fortified (recovery %)
NE	80	1.6	99.01
	100	2	101.00
	120	2.4	100.57
DA	80	3.2	101.44
	100	4	101.23
	120	4.8	99.69
5HT	80	2.4	98.25
	100	3	101.99
	120	3.6	100.87

**Table 5:** Within-run and between-run precision and accuracy results of NAR in rat plasma.

NAR	Parameters	Precision (CV %)		Accuracy	
		Intraday	Interday	Unfortified (µg/ml)	Fortified (Recovery %)
	LLOQ	2.78	2.70	0.2	100.30
	LQC	1.61	1.19	0.5	101.77
	MQC	2.52	2.69	1.5	101.12
	HQC	2.13	1.71	10	98.60

Lower limit of quantification (LLOQ); low quality control; mid quality control (MQC); high quality control (HQC).

### Pharmacokinetic study

**Figure 11:**
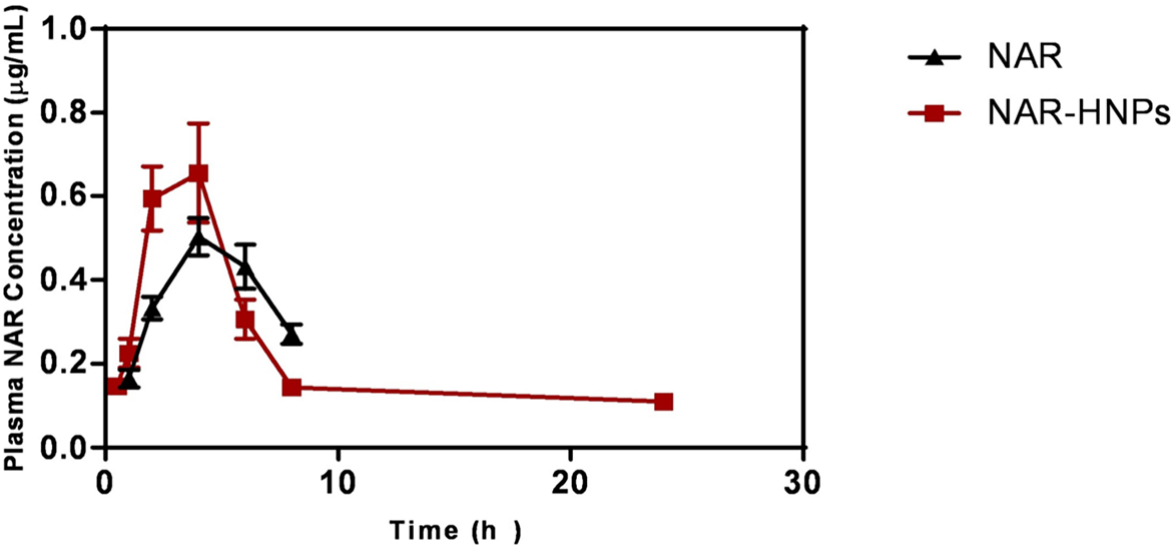
Plasma concentration–time curve of NAR after oral injection of NAR and NAR-HNPs.

Figure 11 depicts NAR’s mean plasma concentration–time profile after oral administration of the pure drug NAR and NAR-HNPs at doses of 50 mg/kg. Table 6 represents the pharmacokinetic analysis. At first glance, it is evident that free NAR is rapidly absorbed and eliminated with t_1/2_ value 4.496 ± 0.2065 h and MRT was 8.326 ± 0.272 h. Free NAR could be detected in plasma for up to 8 h.

**Table 6:** Pharmacokinetic parameters of NAR and NAR-HNPs in normal rats.

Treatment	Parameters								
	t _½_ (h)	λz (h^−1^)	C _max_ (µg/ml)	T _max_ (h)	AUC _0-∞_ (µg ml h^−1^)	MRT (h)	AUMC _0-∞_ (µg ml h^−1^)	V_z_/F (mg/kg) (µg/ml)	CL/F (mg/kg) (µg/ml)
NAR (50 mg/kg)	4.496 ± 0.207	0.154 ± 0.007	0.504 ± 0.026	4 ± 0.0	4.569 ± 0.217	8.326 ± 0.272	38.15 ± 0.287	71.12 ± 3.202	10.97 ± 0.555
NAR-HNPs (50 mg/kg)	10.26* ± 1.21	0.695* ± 0.009	0.670 ± 0.067	4 ± 0.0	6.897* ± 0.281	15.56* ± 1.784	108.3* ± 15.91	106.6* ± 9.15	7.268* ± 0.3028

Results are stated as mean ± S.E.M. (n = 6)* vs corresponding NAR group. NAR, naringenin; NAR-HNPs, naringenin-loaded hybridized nanoparticles; t _½_, elimination half-life; λz, elimination rate constant; C _max_, maximum plasma concentration; t_max_, time to reach C _max_; AUC _0-∞_, area under the plasma concentrations-time curve; MRT, mean residence time; AUMC_0-∞_, area under the first moment curve; V_z_/F, volume of distribution; CL/F, clearance.

After oral administration of NAR-HNPs, C_max_ were approximately 0.6697 ± 0.0673 µg/ml observed at 4 h in normal rats. It is quite evident that NAR-HNPs had faster absorption compared to free NAR. The increase in AUC by about 50% and the prominent increase in AUMC by about 185% revealed increased bioavailability. Peak serum concentrations of NAR-HNPs increased by about 33% compared to free NAR.

t_1/2_ of NAR-HNPs was prolonged about twofold than that of free NAR (10.26 ± 1.21 h for NAR-HNPs and 4.496 ± 0.2065 h for free NAR) and MRT was doubled (15.56 vs 8.326 h) indicating that free NAR was eliminated more slowly for NAR-HNPs than free Nar. NAR could be detected up to 24 h after oral administration of NAR-HNPs. Moreover, the increase of AUC_0-∞_ and AUMC_0-∞_ indicated that bioavailability was enhanced after administration of NAR-HNPs. The volume of distribution was about 50% higher.

## Body weight alterations in rats administered NAR and NAR-HNPs in STZ-induced diabetes

**Figure 12:**
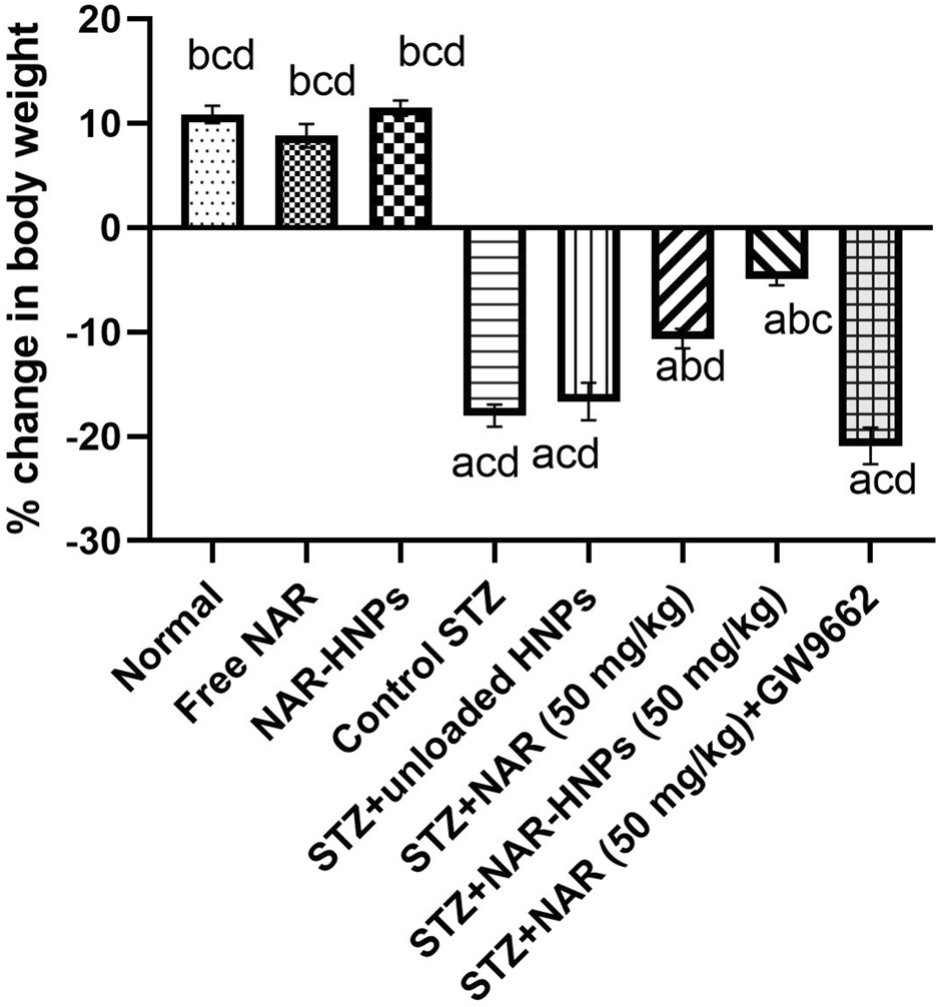
Effect of NAR and NAR-HNPs on body weight changes in diabetic rats. Results are stated as mean ± SEM. ^a^vs normal group, ^b^vs control STZ group, ^c^vs STZ + NAR (50 mg/kg), ^d^vs STZ + NAR-HNPs (50 mg/kg) at *p* < 0.05. NAR, naringenin; STZ, streptozotocin; NAR-HNPs, naringenin- loaded hybridized nanoparticles.

Figure 12 demonstrates that the body weight of diabetic rats decreased by 18.01%, whereas NAR and NAR-HNPs reduced body weight gain by 10.63% and 4.92%, respectively (F = 133.8, *p* < 0.0001). GW9662 administration to NAR-treated diabetic rats markedly reduced body weight by 20.90% (F = 133.8, *p* < 0.0001). There was no significant change within free NAR (F = 133.8; *p* = 0.9217), and NAR-HNPs (F = 133.3; *p* > 0.9999) from normal group. No remarkable change was detected between diabetic and diabetic unloaded HNPs rats (F = 133.8; *p* = 0.9912).

## Behavioral changes in rats administered NAR and NAR-HNPs in STZ-induced diabetes

**Figure 13:**
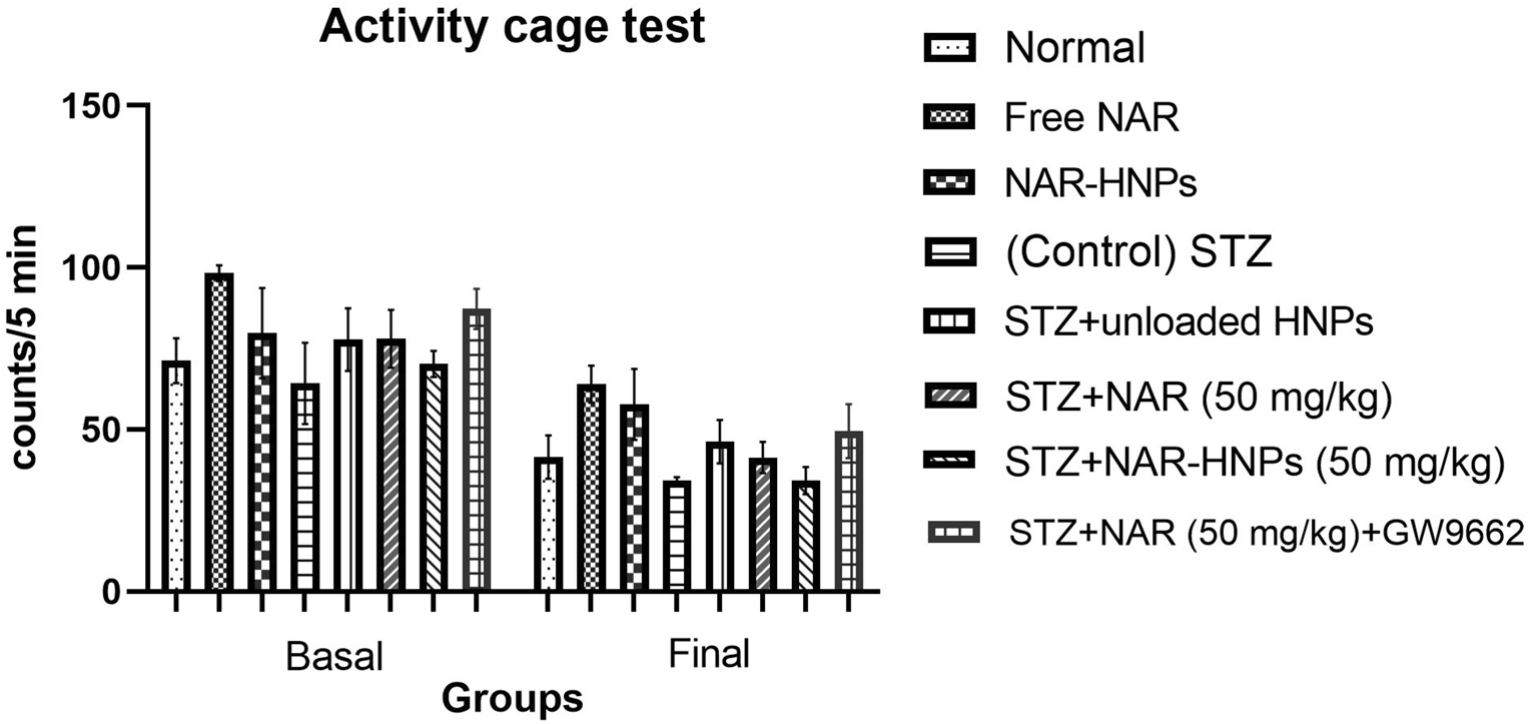
Effect of NAR and NAR-HNPs on spontaneous locomotor activity in diabetic rats subjected to activity cage test. Results are stated as mean ± SEM. NAR, naringenin; STZ, streptozotocin; NAR-HNPs, naringenin- loaded hybridized nanoparticles.

No remarkable difference was noticed between the basal and the correspondent final activity counts (F = 67.3, *p* < 0.0001), nor between the final activity counts of different groups of rats in the activity cage test (F = 3.452, *p* = 0.0022) (Fig. 13).

**Figure 14:**
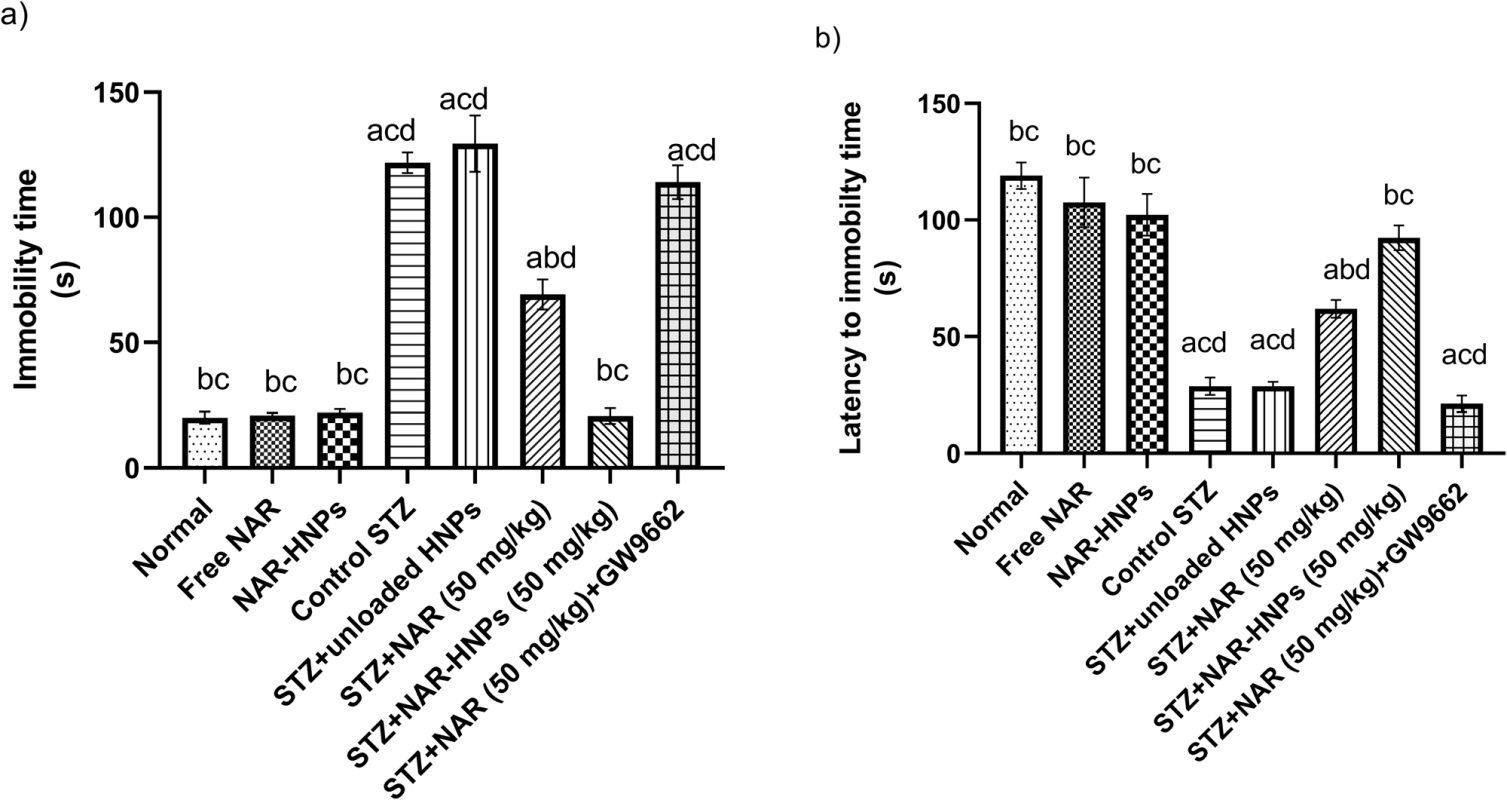
Effect of NAR and NAR-HNPs on (**a**) immobility time, (**b**) latency to immobility time on diabetic rats subjected to FST. Results are stated as mean ± SEM. ^a^vs normal group, ^b^vs control STZ group, ^c^vs STZ + NAR (50 mg/kg), ^d^vs STZ + NAR-HNPs (50 mg/kg) at *p* < 0.05. NAR, naringenin; STZ, streptozotocin; NAR-HNPs, naringenin- loaded hybridized nanoparticles.

As shown in Fig. 14a, the immobility time of diabetic rats was significantly longer than that of normal rats by 6.08 times (F = 82.77, *p* < 0.0001). However, NAR and NAR-HNPs decreased the immobility time by 0.57 and 0.17 times, respectively (F = 82.77, *p* < 0.0001). In comparison to diabetic rats treated with NAR, the addition of GW9662 to NAR-treated rats increased immobility time by 1.65 fold (F = 82.77, *p* < 0.0001). Free NAR (F = 82.77; *p* = 0.9077), and NAR- HNPs [F = 82.77; *p* = 0.7954] groups exhibited no significant change from normal group. There was no discernible difference between diabetic and unloaded diabetic HNPs rats (F = 82.77; *p* = 0.3281).

As shown in Fig. 14b, Diabetic rats significantly shortened latency time to 0.24 fold [F = 43.02, *p* < 0.0001] of the normal group, treatment of diabetic rats with NAR and NAR-HNPs significantly prolonged latency time to 2.16 (F = 43.02, *p* = 0.0003) and 3.22 fold (F = 43.02, *p* < 0.0001), correspondingly. In comparison to diabetic rats treated with NAR, diabetic rats treated with NAR-HNPs had a 1.49-fold increase in latency time (F = 43.02, *p* = 0.0008), whereas the addition of GW9662 to NAR-treated rats shortened latency time by 0.34-fold [F = 43.02, *p* < 0.0001]. Free NAR (F = 43.02; *p* = 0.1876) and NAR- HNPs (F = 43.02; *p* = 0.0566) groups exhibited no significant change as compared to normal group. There was no difference between diabetic and diabetic unloaded HNPs rats (F = 43.02; *p* = 0.9979).

## Levels of blood glucose and serum insulin in rats administered NAR and NAR-HNPs in STZ-induced diabetes

Table 7 reveals that diabetic rats manifested a dramatic elevation in blood glucose level to 4.81 fold (F = 56.65; *p* < 0.0001), and a decline in serum insulin to 0.59 fold (F = 8.18; *p* = 0.0005) as compared to normal rats. In the meantime, treatment with NAR and NAR-HNPs significantly decreased blood glucose levels by 0.80 fold (F = 56.65, *p* = 0.0067) and 0.43 fold (F = 56.65; *p* < 0.0001), respectively, and significantly increased serum insulin levels by 1.88 fold and 1.88 fold (F = 8.18; *p* < 0.0001), respectively as compared to diabetic rats. Combined administration of GW9662 to diabetic rats treated with NAR significantly increased blood glucose level by 1.22 fold (F = 56.65; *p* = 0.0184) and significantly decreased serum insulin by 0.58 fold (F = 8.18; *p* = 0.0001) as compared to diabetic rats. Whereas co-administration of NAR-HNPs to diabetic rats reduced blood glucose level to 0.54-fold (F = 56.65, *p* < 0.0001) in comparison to NAR-treated diabetic rats. There was no significant change within free NAR and NAR-HNPs groups in blood glucose (F = 56.65; *p* = 0.9400); (F = 8.18; *p* = 0.5505) and serum insulin (F = 56.65; *p* = 0.9116]; (F = 8.18; *p* = 0.2242) from normal group, respectively. There was no difference among diabetic and diabetic unloaded HNPs rats in blood glucose (F = 56.65; *p* = 0.9400) and serum insulin (F = 8.18; *p* = 0.9238) from normal rats, respectively.

**Table 7:** Effect of NAR and NAR-HNPs on blood glucose and serum insulin levels in experimental rats.

Groups	Parameters	
	Blood glucose (mg/dl)	Serum insulin (mU/l)
Normal	122.33^bc^ ± 7.56	64.30^b^ ± 4.03
Free NAR	119.17^bc^ ± 9.51	60.06^b^ ± 4.35
NAR-HNPs	117.67^bc^ ± 6.89	55.60^b^ ± 1.53
Control STZ	588.00^acd^ ± 9.48	37.77^acd^ ± 5.58
STZ + unloaded HNPs	591.11^acd^ ± 6.45	37.09 ^acd^ ± 5.15
STZ + NAR (50 mg/kg)	468.50^abd^ ± 54.53	71.13^b^ ± 3.59
STZ + NAR-HNPs (50 mg/kg)	253.83^abc^ ± 53.37	70.52^b^ ± 8.89
STZ + NAR (50 mg/kg) + GW9662	571.17^acd^ ± 28.83	41.16^acd^ ± 3.40

Results are stated as means ± SEM (n = 6), ^a^ vs normal group, ^b^ vs control STZ group, ^c^ vs STZ + NAR (50 mg/kg), ^d^ vs STZ + NAR-HNPs (50 mg/kg) at *p* < 0.05. NAR, naringenin; STZ, streptozotocin; NAR-HNPs, naringenin- loaded hybridized nanoparticles.

## Cortical biochemical changes in rats administered NAR and NAR-HNPs in STZ-induced diabetes

**Figure 15:**
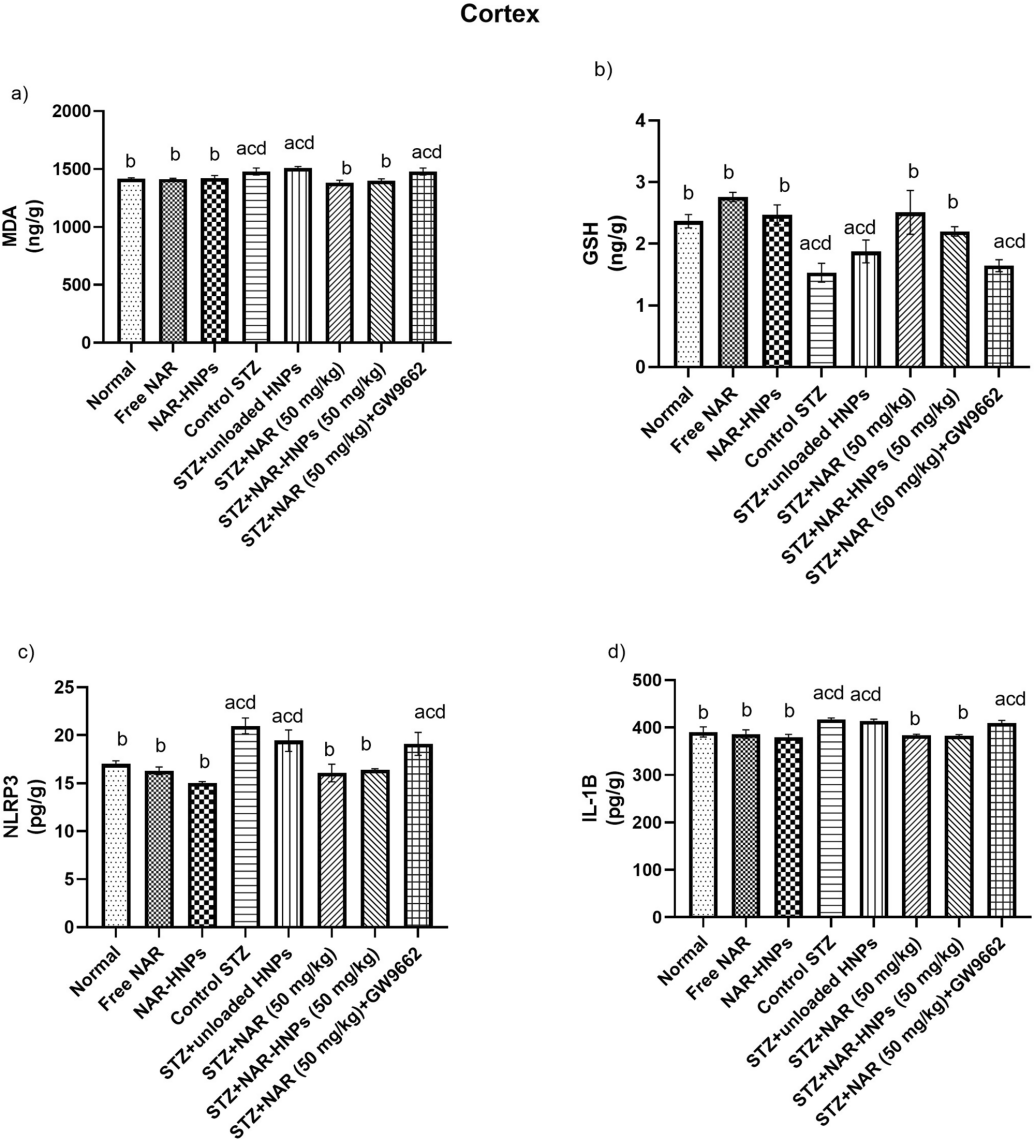
Effect of NAR and NAR-HNPs on (**a**) GSH, (**b**) MDA, (**c**) NLRP3, (**d**) IL-1β cortical contents in diabetic rats. Results are stated as mean ± SEM. ^a^vs normal group, ^b^vs control STZ group, ^c^vs STZ + NAR (50 mg/kg), ^d^vs STZ + NAR-HNPs (50 mg/kg) at *p* < 0.05. NAR, naringenin; STZ, streptozotocin; NAR-HNPs, naringenin- loaded hybridized nanoparticles.

As illustrated in Fig. 15a–d, diabetic rats significantly elevated cortical MDA to 1.04 fold (F = 4.88; *p* = 0.038), lowered GSH to 0.65 fold (F = 9.01; *p* = 0.0016), elevated NLRP3, as well as IL-1ꞵ contents to 1.23 fold (F = 7.51; *p* = 0.0006) and 1.07 fold (F = 6.20; *p* = 0.004), respectively as compared to normal rats.

Giving oral doses of NAR and NAR-HNPs to diabetic rats significantly reduced cortical MDA content to 0.93 fold (F = 4.88; *p* = 0.0019) and 0.95 fold (F = 4.88; *p* = 0.008), respectively; elevated GSH to 1.64 fold (F = 9.01; *p* = 0.0003) and 1.44 fold (F = 9.01; *p* = 0.0094) respectively, reduced NLRP-3 to 0.77 fold and 0.78 fold (F = 7.51; p < 0.0001), respectively and reduced IL-1ꞵ content to 0.92 fold (F = 6.20; *p* = 0.0004) and 0.92 fold (F = 6.20; *p* = 0.0003), respectively as compared to diabetic rats.

Administration of GW9662 blocked the effect of NAR on diabetic rats elevated MDA to 1.07 fold (F = 4.88; *p* = 0.0019), reduced GSH to 0.66 fold (F = 9.01; *p* = 0.0011), elevated NLRP-3 to 1.19 fold (F = 7.51; *p* = 0.0068) and IL-1ꞵ to 1.07 fold (F = 6.20; *p* = 0.0058) in comparison to NAR rats. Both free NAR and NAR-HNPs showed no significant change in cortical contents of MDA (F = 4.88; *p* = 0.9536); (F = 4.88; *p* = 0.9184), GSH (F = 6.42; *p* = 0.1187); (F = 6.42; *p* = 0.6797), NLRP-3 (F = 7.51; *p* = 0.5132); (F = 7.51; *p* = 0.0674) and IL-1ꞵ (F = 6.201; *p* = 0.5921) (F = 6.201; *p* = 0.2004) as compared to normal group, respectively. There was no significant between diabetic and diabetic unloaded HNPs rats in the cortical contents of MDA (F = 4.88; *p* = 0.3092), GSH (F = 6.43; *p* = 0.1686), NLP-3 (F = 0.1542; *p* = 0.1542) and IL-1ꞵ (F = 6.201; *p* = 0.6489).

## Hippocampal biochemical changes in rats administered NAR and NAR-HNPs in STZ-induced diabetes

**Figure 16:**
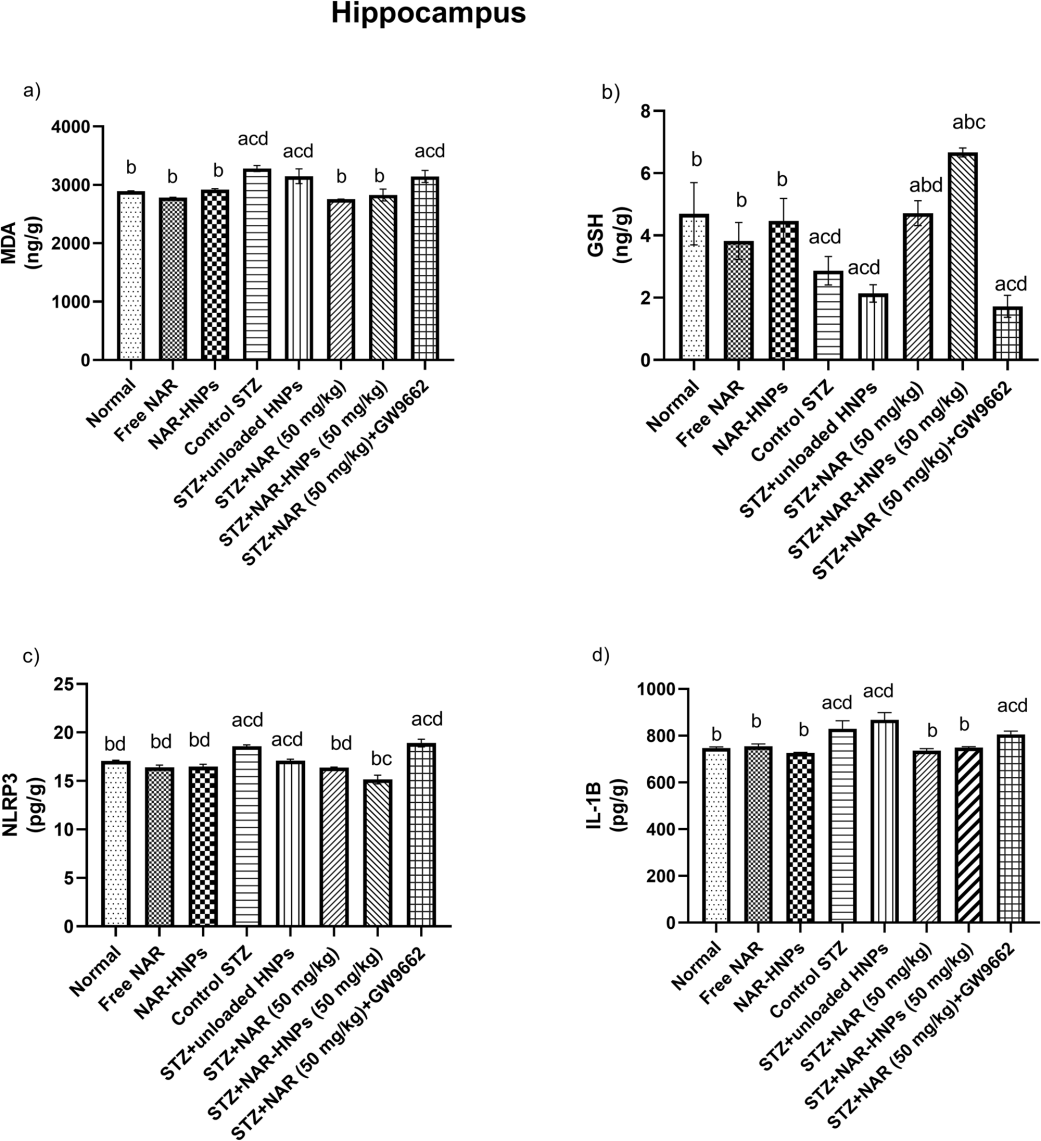
Effect of NAR and NAR-HNPs on (**a**) GSH, (**b**) MDA, (**c**) NLRP3, (**d**) IL-1β hippocampal contents in diabetic rats. Results are stated as mean ± SEM. ^a^vs normal group, ^b^vs control STZ group, ^c^vs STZ + NAR (50 mg/kg), ^d^vs STZ + NAR-HNPs (50 mg/kg) at *p* < 0.05. NAR, naringenin; STZ, streptozotocin; NAR-HNPs, naringenin- loaded hybridized nanoparticles.

Figure 16a–d depicts that the STZ group significantly increased hippocampal MDA by 1.13 fold (F = 7.65; *p* = 0.0004), decreased GSH by 0.61 fold (F = 8.46; *p* = 0.0248), and increased NLRP3 and IL-1β contents by 1.09 fold (F = 23.49; *p* = 0.0002) and 1.11fold (F = 8.25; *p* = 0.0023), respectively as compared to normal rats.

Treatment of diabetic rats with NAR and NAR-HNPs significantly reduced hippocampal MDA content to 0.84 and 0.86 fold (F = 7.65; *p* < 0.0001), respectively; elevated GSH content to 1.64 fold (F = 8.46; *p* = 0.0235) and 2.32 fold (F = 8.46; *p* < 0.0001), respectively; reduced NLRP3 content to 0.88 fold and 0.82 fold (F = 23.49; *p* < 0.0001), correspondingly and reduced IL-1ꞵ content to 0.89 fold (F = 8.25; *p* = 0.0006) and 0.90 fold (F = 8.25; *p* = 0.0027) , respectively as compared to diabetic rats.

Co-administration of GW9662 significantly reduced GSH content to 0.36 fold (F = 8.46; *p* = 0.0005); increased hippocampal content of MDA to 1.14 fold (F = 7.65; *p* = 0.0004); NLRP3 to 1.15 fold (F = 23.49; *p* < 0.0001) and IL-1ꞵ to 1.09 fold (F = 8.25; *p* = 0.0084) in relation to NAR-treated diabetic rats.

NAR-HNPs significantly elevated GSH content to 1.41 fold (F = 8.46;* p* = 0.0017) and decreased NLRP3 content to 0.92 fold (F = 23.49; *p* = 0.0013) in comparison to NAR-treated diabetic rats.

Both free NAR and NAR-HNPs showed remarkable significant difference in hippocampal contents of MDA (F = 0.77; *p* = 0.3010); (F = 0.77; *p* = 0.7795), GSH (F = 8.459; *p* = 0.2731); (F = 8.459; *p* = 0.7766), NLRP-3 (F = 23.49; *p* < 0.0001); (F = 23.49; *p* < 0.0001) and IL-1ꞵ (F = 8.252; *p* = 0.7814) (F = 8.252; *p* = 0.4179) as compared to normal group, respectively. There was no significant between diabetic and diabetic unloaded HNPs rats in the cortical contents of MDA (F = 0.77; *p* = 0.1981), GSH (F = 8.46; *p* = 0.3575), NLRP3 (F = 23.49; *p* = 0.1542) and IL-1ꞵ (F = 8.25; *p* = 0.1325).

## Cortical monoamines and metabolites in rats administered NAR and NAR-HNPs in STZ-induced diabetes

Regarding cortical neurotransmitters and metabolites, diabetic rats revealed a significant reduction in NE to 0.53 fold (F = 27.72; *p* < 0.0001), 5-HT to 0.65 fold (F = 6.70; *p* = 0.0007), DA to 0.68 fold (F = 12.91; *p* < 0.0001), respectively. When compared to normal rats, the diabetic group increased 5-HTAA to 1.29 fold (F = 17.22; *p* = 0.0063), DOPAC to 1.3 fold (F = 17.14; *p* < 0.0001) (Table 8).

**Table 8:** Effect of NAR and NAR-HNPs on cortical monoamines and metabolites in experimental rats.

Groups	Parameters				
	Cortex (μg/g)				
	NE	5-HT	5-HTAA	DA	DOPAC
Normal	0.34^b^ ± 0.01	0.40^b^ ± 0.05	0.07^b^ ± 0.00	0.63^b^ ± 0.01	0.10^b^ ± 0.00
Free NAR	0.36^b^ ± 0.03	0.37^b^ ± 0.02	0.07^b^ ± 0.00	0.58^b^ ± 0.02	0.10^b^ ± 0.01
NAR-HNPs	0.31^b^ ± 0.02	0.36^b^ ± 0.03	0.07^b^ ± 0.00	0.55^b^ ± 0.01	0.10^b^ ± 0.00
Control STZ	0.18^acd^ ± 0.01	0.26 ^acd^ ± 0.01	0.09 ^acd^ ± 0.00	0.43 ^acd^ ± 0.02	0.13 ^acd^ ± 0.01
STZ + unloaded HNPs	0.18 ^acd^ ± 0.01	0.27 ^acd^ ± 0.03	0.10 ^acd^ ± 0.01	0.42 ^acd^ ± 0.04	0.13 ^acd^ ± 0.01
STZ + NAR (50 mg/kg)	0.25^abd^ ± 0.02	0.37^b^ ± 0.03	0.07 ^bd^ ± 0.00	0.57 ^bd^ ± 0.04	0.11^bd^ ± 0.00
STZ + NAR-HNPs (50 mg/kg)	0.37^bc^ ± 0.01	0.44^b^ ± 0.01	0.08 ^bc^ ± 0.00	0.68^bc^ ± 0.04	0.10 ^bc^ ± 0.00
STZ + NAR (50 mg/kg) + GW9662	0.18^acd^ ± 0.00	0.27 ^acd^ ± 0.00	0.09 ^acd^ ± 0.01	0.41^acd^ ± 0.01	0.14 ^acd^ ± 0.00

Results are stated as means ± SEM (n = 6), ^a^ vs normal group, ^b^ vs control STZ group, ^c^ vs STZ + NAR (50 mg/kg), ^d^ vs STZ + NAR-HNPs (50 mg/kg) at *p* < 0.05. NAR, naringenin; STZ, streptozotocin; NAR-HNPs, naringenin- loaded hybridized nanovesicles.

Diabetic rats treated with NAR and NAR-HNPs showed a significant increase in cortical contents of; NE to 1.39 fold (F = 27.72; *p* = 0.0015) and 2.06 fold (F = 27.72; *p* < 0.0001) respectively; 5-HT to 1.42 fold (F = 6.70; *p* = 0.0053) and 1.69 fold (F = 6.7, *p* < 0.0001) respectively; DA to 1.33 fold (F = 12.91; *p* = 0.0014) and 1.58 fold (F = 12.91; *p* < 0.0001) respectively. NAR and NAR-HNPs treated diabetic rats significantly reduced 5-HTAA to 0.78 fold (F = 17.22; *p* < 0.0001) and 0.89 fold (F = 17.22; *p* = 0.039); DOPAC to 0.85 fold (F = 17.14; *p* = 0.039) and 0.77 fold (F = 1714; *p* < 0.0001) in comparison with diabetic rats.

Combining the administration of GW9662 to diabetic rats treated with NAR significantly reduced NE content by 0.72 fold (F = 27.72; *p* = 0.0045), 5-HT content by 0.73 fold (F = 6.70; *p* = 0.0065), and DA content by 0.72 fold (F = 12.91; *p* = 0.0002), whereas it increased 5-HTAA content by 1.28 fold (F = 17.22; *p* < 0.0001) and DOPAC content by 1.27 fold (F = 17.14; *p* < 0.0001) relative to the NAR-treated group.

When compared to NAR, diabetic rats treated with NAR-HNPs had 1.48 fold higher NE (F = 27.72; *p* < 0.0001), 1.19 fold higher DA (F = 12.91; *p* = 0.0096), and 0.91 fold lower DOPAC content (F = 17.14; *p* = 0.0399) (Table 8).

## Hippocampal monoamines and metabolites in rats administered NAR and NAR-HNPs in STZ-induced diabetes

Table 9 reveals that, STZ administered rats exhibited a significant decline in hippocampal contents of NE to 0.69 fold (F = 30.54; *p* < 0.0001), 5-HT to 0.58 fold (F = 12.27, *p* < 0.0001), DA to 0.67 fold (F = 15.77; *p* < 0.0001) and an increase in contents of 5-HTAA to 1.54 fold (F = 12.15; *p* < 0.0001) and DOPAC to 1.35 fold (F = 12.99; *p* < 0.0001) as compared to normal rats.

**Table 9:** Effect of NAR and NAR-HNPs on hippocampal monoamines and metabolites in experimental rats.

Groups	Parameters				
	Hippocampus (μg/g)				
	NE	5-HT	5-HTAA	DA	DOPAC
Normal	0.62^b^ ± 0.02	0.62^b^ ± 0.05	0.13^b^ ± 0.01	1.50^b^ ± 0.05	0.26^b^ ± 0.02
Free NAR	0.60^b^ ± 0.01	0.62^b^ ± 0.06	0.15^b^ ± 0.01	1.35^b^ ± 0.02	0.25^b^ ± 0.00
NAR-HNPs	0.57^b^ ± 0.02	0.71^b^ ± 0.07	0.14^b^ ± 0.00	1.53^b^ ± 0.07	0.26^b^ ± 0.02
Control STZ	0.43^acd^ ± 0.02	0.36 ^acd^ ± 0.01	0.20 ^acd^ ± 0.00	1.00 ^acd^ ± 0.07	0.35 ^acd^ ± 0.01
STZ + unloaded HNPs	0.40 ^acd^ ± 0.02	0.40 ^acd^ ± 0.03	0.19 ^acd^ ± 0.01	1.09 ^acd^ ± 0.01	0.34 ^acd^ ± 0.00
STZ + NAR (50 mg/kg)	0.53^abd^ ± 0.00	0.54 ^bd^ ± 0.01	0.17 ^abd^ ± 0.01	1.32 ^abd^ ± 0.05	0.27^bd^ ± 0.02
STZ + NAR-HNPs (50 mg/kg)	0.62^bc^ ± 0.01	0.70 ^bc^ ± 0.02	0.14^bc^ ± 0.01	1.70^abc^ ± 0.10	0.23 ^bc^ ± 0.01
STZ + NAR (50 mg/kg) + GW9662	0.43 ^acd^ ± 0.01	0.39 ^acd^ ± 0.01	0.20 ^acd^ ± 0.01	1.04 ^acd^ ± 0.08	0.34 ^acd^ ± 0.01

Results are stated as means ± SEM (n = 6), ^a^ vs normal group, ^b^ vs control STZ group, ^c^ vs STZ + NAR (50 mg/kg), ^d^ vs STZ + NAR-HNPs (50 mg/kg) at *p* < 0.05. NAR, naringenin; STZ, streptozotocin; NAR-HNPs, naringenin- loaded hybridized nanoparticles.

Oral administration of NAR and NAR-HNPs strikingly elevated hippocampal contents of NE to 1.23 fold (F = 30.54; *p* = 0.0016) and 1.44 fold (F = 30.54; *p* < 0.0001), correspondingly; 5-HT to 1.5 fold (F = 12.27; *p* = 0.0038) and 1.94 fold (F = 12.27; *p* < 0.0001), correspondingly; DA to 1.32 fold (F = 15.77; *p* = 0.0011), and 1.70 fold (F = 15.77; *p* < 0.0001), respectively and reduced 5-HTAA to 0.85 fold (F = 12.15; *p* = 0.009) and 0.70 fold (F = 12.15;* p* < 0.0001), respectively; and reduced DOPAC to 0.77 fold (F = 12.99; *p* = 0.0001) and 0.66 fold (F = 12.99; *p* < 0.0001), respectively in relation to diabetic rats.

Co-treatment of diabetic rats with NAR and GW9662 significantly diminished hippocampal contents of NE to 0.81 fold (F = 30.54; *p* = 0.0012), 5-HT to 0.72 fold (F = 12.27; *p* = 0.0146), DA to 0.79 fold (F = 15.77; *p* = 0.0038) and elevated 5-HTAA to 1.18 fold (F = 12.15; *p* = 0.0049) and DOPAC to 1.26 fold (F = 12.99; *p* = 0.0015) relative to the NAR group.

NAR-HNPs treatment significantly elevated NE to 1.17 fold (F = 30.54; *p* = 0.0202), 5-HT to 1.30 fold (F = 12.27; *p* = 0.0081), DA to 1.29 fold (F = 15.77; *p* = 0.0002) and reduced 5-HTAA to 0.82 fold (F = 12.15; *p* = 0.0148) and DOPAC to 0.85 fold (F = 12.99; *p* = 0.015) in comparison with NAR group (Table 9).

### Cortical and hippocampal PPAR-γ gene expression in rats administered NAR and NAR-HNPs in STZ-induced diabetes.

PPAR*-*γ gene expression was investigated in cortical and hippocampal tissues by RT-PCR, as illustrated in Table 10. Diabetic rats significantly reduced cortical and hippocampal *PPAR*-*γ* gene expression as compared to normal rats with 3.9 (F = 95.60; *p* < 0.0001) and 5.08 fold-change (F = 76.78; *p* < 0.0001), respectively. Oral administration NAR and NAR-HNPs up-regulated the *PPAR*-*γ* gene expression in cortical with 2.61 and 2.55 fold-change (F = 95.60; *p* < 0.0001), respectively. Treatments with NAR and NAR-HNPs up-regulated the PPAR*-*γ gene expression in hippocampal with 4.70 and 4.50 fold-change (F = 76.78; *p* < 0.0001), respectively.

**Table 10:** Relative expression of PPAR-γ genes in cortex and hippocampus of different experimental rats.

Groups	Parameters	
	Relative expression of PPARγ (Fold-change)	
	Cortex	Hippocampus
Normal	4.03^b^ ± 0.11	5.23^b^ ± 0.37
Free NAR (50 mg/kg)	3.38^b^ ± 0.18	4.96^b^ ± 0.10
NAR- HNPs (50 mg/kg)	3.53^b^ ± 0.20	4.77^b^ ± 0.11
Control STZ (50 mg/kg)	1.01^acd^ ± 0.09	1.03^acd^ ± 0.17
STZ + unloaded HNPs	1.39^acd^ ± 0.20	1.38^acd^ ± 0.22
STZ + NAR (50 mg/kg)	2.63^ab^ ± 0.02	4.83^b^ ± 0.31
STZ + NAR-HNPs (50 mg/kg)	2.57^ab^ ± 0.08	4.62^b^ ± 0.18
STZ + NAR (50 mg/kg) + GW9662	1.32^acd^ ± 0.11	1.34^acd^ ± 0.18

The mRNA expression PPAR-γ is normalized with housekeeping gene (GAPDH), values are stated as means ± SEM, n = 3 per group (all samples run in duplicate and no template control were included in all runs). ^a^ vs normal group, ^b^ vs control STZ group, ^c^ vs STZ + NAR (50 mg/kg), ^d^ vs STZ + NAR-HNPs (50 mg/kg) at *p* < 0.05. NAR, naringenin; STZ, streptozotocin; NAR-HNPs, naringenin- loaded hybridized nanoparticles.

Co-administration of GW9662 to NAR-treated diabetic rats abolished the effect of NAR on cortical (F = 95.60; *p* < 0.0001) and hippocampal (F = 76.78; *p* < 0.0001) PPAR-*γ* gene expression.

## Discussion

The current study generates new evidence on the anti-depressant effect of NAR and NAR-HNPs on STZ-induced diabetic rats. To the best of the authors’ knowledge this is the first study to demonstrate the role of PPARγ/NLRP3 pathway, cortical and hippocampal neurotransmitters in the anti-depressant effect of NAR and NAR-HNPs in diabetic rats. To uncover the involvement of PPARγ in the anti-depressant effect of NAR and NAR-HNPs, the effect of GW9962 was investigated. GW9962 was used to emphasize the involvement of PPARγ in the anti-depressant effect of NAR and NAR-HNPs. The current findings prove that NAR-HNPs is more effective than NAR in mitigating depression in diabetic rats.

As depicted from this study, HNPs containing lipoid S45 displayed high EE% (>95%) compared to HNPs lacking lipoid S45. It was previously reported that increased drug encapsulation is more strongly impacted by lecithin incorporation^65^. This occurs when the phosphate groups in the negatively charged polar lecithin combine with the NH^3+^ groups of CS, creating nanoparticles. Moreover, the increase in viscosity at higher concentrations, which causes the creation of larger droplets and ultimately larger nanoparticles, could be responsible for the observed increase in size with increasing CS concentration from 0.2 to 0.4% w/v^66^. Moreover, the interaction between the negatively charged lipoid S45 and the positively charged CS increases the diameter of HNPs^67,68^. The highly positive values for ZP observed for the HNPs prepared with lipoid S45 are also attributed to the electrostatic interaction of chitosan and lipids (Table 2). This finding is in harmony with prior study which demonstrates that during preparing the formulation of lecithin/chitosan hybrid nanoparticles, the cationic charge of chitosan dominate the anionic charge of lipoid^69^. At the same time, this recorded high ZP values play an important role on the stability of nanoparticles as particles in suspension will tend to reject each other and not agglomerate if all of the particles have the lowest negative ZP (above or equal to −30 mV) and the highest positive zeta potential (above or equal to +30 mV)^62^. As known, a PDI value less than 0.3 is regarded as acceptable, indicating a homogeneous population of nanoformulations^70,71^, which has already checked in NAR-HNP5 and NAR-HNP6. The lowest PDI value for NAR-HNP6 (0.21±0.02) is a good indication for the homogeneity of the system and proves a good distribution and little fluctuation in the PS^72^.

Regarding the *in vitro* release study, it is clear that increasing the concentration of chitosan from 0.2 to 0.4 % w/v decreases the release of NAR from the NAR-HNPs which can be returned to the interaction between the NAR and the CS resulting in retardation of the drug release. The more sustained release observed in NAR-HNP7 and NAR-HNP8 can be returned to the high CS concentration (0.4%w/v) beside the presence of lipoid S45 which exhibit a greater stabilization resulting in more release retardation^73^. On the other hand, NAR-HNP6 showed 75.2±2.24% NAR released after 24 h which makes it a good candidate to be chosen as the best formulation realizing the highest percent of NAR released compared to the other tested NAR-HNPs.

The NAR-HNP6 DSC thermogram revealed that there were no typical peaks for either NAR or CS, proving that the drug had been transformed into an amorphous form and that it had been incorporated into the NPs. As well, the TEM study shows how the particles are separated from one another which is a good indication of their stability^74^. The inner bright portion of the NPs represents the polymeric core encapsulating the drug, which is surrounded by a dark shell representing the lipid^69^. NAR-HNP6 exhibited an acceptable oral pH of 4.9±0.02 and displayed a physicochemical stability at 4 °C.

The pharmacokinetics evaluation showed that the novel formulation NAR-HNPs possesses superior absorption and elimination characteristics than free NAR. This was evident in the increase in C_max_ and substantial prolongation of half-life and λz compared to free NAR.

Additionally, the increase of MRT points to increased drug retention in the systemic circulation; hence, providing an extended drug acting time. The observed increase in bioavailability, in terms of enhanced AUC and AUMC, in addition to enhanced MRT could be attributed to nanonisation that increases solubilization, and dissolution rate, thereby improving absorption in GIT and permitting efficient uptake in the intestine and prolonging drug retention at the absorption site^75^.

CS coating improves drug retention capability and cellular uptake through enhanced mucoadhesion to the mucin layer that delineates the superficial layer of enterocytes and M layers located in the intestine^76^. CS also improves oral absorption through inhibiting permeation-glycoprotein (P-gp) activity. P-gp is a membrane transporter that actively pumps drugs out of the enterocytes leading to decreased intestinal permeability and decreased oral absorption and bioavailability. By inhibiting P-gp activity, residence time of the drugs at active sites is prolonged, and hence absorption efficiency is increased^77,78^. GMO hinders enzymatic degradation during absorption and therefore reduces first-pass metabolism which is crucial for oral absorption^79,80^. In addition, entrapment into GMO enhances solubility of insoluble drug and thus facilitates their transport across cell membrane and consequently their oral absorption and oral bioavailability^81^. GMO also improve oral absorption via lowering P-gp activity^82^. The increase in the volume of distribution points to a higher distribution capacity of the formulation. Polymeric nanoparticles, have been found to be a promising solution to address a number of conventional issues, including low solubility, increased stability, a sharper size distribution, sustained and controlled release profiles, higher encapsulation efficiency for agents that are weakly water soluble, and the potential to improve bioavailability^83^.

In this study, 48 h following a single intraperitoneal administration of STZ in a dose of 50 mg/kg resulted in a marked elevation in blood glucose, body weight loss, depletion in serum insulin in addition to alteration in depressive-related parameters as FST, cortical and hippocampal monoaminergic contents.

The deficiency of monoamine neurotransmitters has emerged as a prominent area of investigation concerning the pathogenesis and treatment of depression. The main neurotransmitters in the brain are NE, 5-HT, and DA. NE is responsible for anxiety, alertness and attention whereas 5-HT affects the human mood and DA is related to motivation and reward^84^. In this study, STZ-induced depletion in cortical and hippocampal NE, 5-HT, and DA contents in addition to elevation in 5-HTAA and DOPAC contents. Those findings demonstrate the successfulness of the current depression-like model.

Moreover, STZ-induced depressive-like behavior was associated with oxidative stress evidenced by elevation in MDA and reduction in GSH, interference with PPAR-γ/NLRP3 pathway witnessed by reduction in cortical and hippocampal PPAR-γ expressions and elevation in both NLRP3 and IL-1β contents. Those findings are in line with prior studies which stated the involvement of oxidative stress and inflammation in the pathophysiology STZ associated depression^85,86^.

Anti-hyperglycemic effect of NAR and NAR-HNPs was witnessed by a reduction in blood glucose, elevation of serum insulin, and attenuation of body weight loss in comparison with diabetic rats and thereby improvement of the general health. As glycemic control plays a pivotal role in combating depression in diabetic patients, the anti-depressant effect of NAR and NAR-HNPs may be partly mediated via alleviation of hyperglycemia in diabetic rats. In line with this finding, NAR attenuated hyperglycemia in experimentally induced diabetic retinopathy and nephropathy in rats^87,88^.

Treatment with NAR and NAR-HNPs did not show any change in locomotor activity in the activity cage test, shortened immobility time and lengthened latency to immobility in FST in comparison with diabetic rats. Those findings reveal that the anti-depressant effect of NAR and NAR-HNPs was independent on changes in motor activity. In accordance, the anti-depressant effect of NAR has been previously reported in chronic unpredictable mild stress rat model^37^. This behavioral amelioration in NAR and NAR-HNPs-treated rats is in line with the noticeable increment in the content of neurotransmitters such as NE, 5-HT, and DA in the cortex and hippocampal region as well as a decrease in the levels of 5-HTAA and DOPAC metabolites in comparison to diabetic rats. In accordance, prior study showed that NAR reduced altered serotonergic, and dopaminergic neurotransmission in Alzheimer’s disease rat model^89^.

NAR and NAR-HNPs anti-oxidant effect was evidenced by combating lipid peroxidation damage shown in the reduction of MDA contents in addition to promoting oxidative defense as revealed in the elevation of GSH contents in the cortex and hippocampus of diabetic rats. Therefore, their anti-depressant effect may be mediated via their anti-oxidant effect. In accordance, NAR exerted an anti-oxidant effect in the olfactory bulbectomy-induced depression model in mice^36^. It is well-established that hyperglycemia provokes oxidative stress and contributes to depression^90^. Therefore, NAR and NAR-HNPs anti-oxidant effect may have resulted from the mitigation of hyperglycemia.

Herein, NAR and NAR-HNPs significantly reduced NLRP3 and thereby IL-1β cortical and hippocampal contents in diabetic rats, revealing that the anti-depressant effect of NAR and NAR-HNPs could be partly linked to its anti-inflammatory effect. It is well-documented that oxidative stress accompanies inflammasome-mediated inflammation in depressed patients. NLRP3 activation results in the production of IL-1β, a pro-inflammatory cytokine that possess a crucial role in the progression of depression^91^. Also, previous studies reported that anti-depressant treatment did not affect TNF-ɑ serum level but reduced IL-1β level, implicating the vitality of IL-1β in the treatment regimen^92^. Therefore, NAR and NAR-HNPs anti-depressant effect could have resulted from altering the oxido-inflammatory response. Consistent with the current finding, NAR showed anti-depressant effect by alleviating inflammatory response in mice subjected to hypoxic stress^35^.

Mounting evidence reported correlation between PPARγ and NLRP proteins^93^. Herein, NAR and NAR-HNPs significantly elevated PPARγ cortical and hippocampal gene expression compared to diabetic rats. Furthermore, GW9662 reversed NAR inhibitory effects on oxidative stress biomarkers and NLRP3 inflammasome activation. The current findings implicate the role of PPAR-γ/NLRP3 pathway in mediating the anti-depressant effect of NAR and NAR-HNPs in diabetic rats. In accordance, NAR increased PPARγ expression in experimental rat models of dementia and stroke^94,95^.

## Conclusion

This study showed that NAR and NAR-HNPs possessed anti-depressant effect in diabetic rats, which could be interposed by mitigation of hyperglycemia, amelioration of oxidative stress and inflammatory response via PPARγ/NLRP3 pathway in addition to activation of monoaminergic system. GW9662 reversed those effects implicating the vital role of PPARγ in mediating NAR anti-depressant effect. NAR-HNPs showed the best anti-depressant effect in diabetic rats. Lipid polymer HNPs made using the self-assembly method demonstrated improved NAR release profile, stability at 4 °C, good drug encapsulation efficiency and bioavailability with the required PS and PDI than free NAR. As the behavioral amelioration of NAR-HNP-treated rats in FST is in line with the distinguished elevations in cortical and hippocampal NE, 5-HT, and DA contents and reduction in 5-HTAA, HVA and DOPAC metabolites compared to NAR-treated rats. Thus, the loading of NAR in HNPs has a potent potential to be investigated as an anti-depressant therapy in DM. Further studies are necessary to elucidate the pathological alterations that may derive the anti- depressant effect of NAR-HNPs in diabetic rats. Clinical studies focusing on long term safety concerns, and delivery challenges are necessary to assess NAR-HNPs' effectiveness as a therapeutic agent for the treatment of depression in diabetic patients. Regarding future scope, it is important to explore the potential for personalized depression treatments by modifying nanoparticle formulations based on individual patient profiles, genetics, and drug responses. Also, it is essential to investigate the use of NAR-HNPs in combination with other anti-depressant drug to enhance treatment outcomes.

### Supplementary Information

Supplementary Information.

## Data Availability

All data generated or analysed during this study are included in this published article.

## References

[1] Abdelkader NF, Elbaset MA, Moustafa PE, Ibrahim SM (2022). Empagliflozin mitigates type 2 diabetes-associated peripheral neuropathy: a glucose-independent effect through AMPK signaling. Arch. Pharm. Res..

[2] Mathur P, Leburu S, Kulothungan V (2022). Prevalence, awareness, treatment and control of diabetes in india from the countrywide national NCD monitoring survey. Front. Public Health.

[3] Sun H (2022). IDF diabetes atlas: Global, regional and country-level diabetes prevalence estimates for 2021 and projections for 2045. Diabetes Res. Clin. Pract..

[4] Wu H (2022). Diabetes and its cardiovascular complications: Comprehensive network and systematic analyses. Front. Cardiovasc. Med..

[5] Le Dinh T (2022). The relationship between depression and multifactorial control and microvascular complications in vietnamese with Type 2 diabetes mellitus aged 30–60 years. Diabetes Metab. Syndr. Obes..

[6] Roy T, Lloyd CE (2012). Epidemiology of depression and diabetes: a systematic review. J. Affect Disord..

[7] Ke Y (2019). Preventive and therapeutic effects of Astaxanthin on depressive-like behaviors in high-fat diet and Streptozotocin-treated rats. Front. Pharmacol..

[8] Paula Farias Waltrick A (2022). Preventive treatment with fish oil facilitates the antidepressant-like effect of antidepressant drugs in type-1 diabetes mellitus rats: Implication of serotonergic system. Neurosci Lett.

[9] Jayaraman R, Subramani S, Sheik Abdullah SH, Udaiyar M (2018). Antihyperglycemic effect of hesperetin, a citrus flavonoid, extenuates hyperglycemia and exploring the potential role in antioxidant and antihyperlipidemic in streptozotocin-induced diabetic rats. Biomed. Pharmacother..

[10] Herman FJ, Pasinetti GM (2018). Principles of inflammasome priming and inhibition: Implications for psychiatric disorders. Brain Behav. Immun..

[11] Wang D (2020). P2X7 receptor mediates NLRP3 inflammasome activation in depression and diabetes. Cell Biosci..

[12] Kim IS, Silwal P, Jo EK (2023). Peroxisome proliferator-activated receptor-targeted therapies: Challenges upon infectious diseases. Cells.

[13] Fu CC (2022). PPARgamma dysfunction in the medial prefrontal cortex mediates high-fat diet-induced depression. Mol. Neurobiol..

[14] Zhou G, Yan M, Guo G, Tong N (2019). Ameliorative effect of berberine on neonatally induced type 2 diabetic neuropathy via modulation of BDNF, IGF-1, PPAR-gamma, and AMPK expressions. Dose Response.

[15] Garg S, Deshmukh VR, Prasoon P (2017). Possible modulation of PPAR-gamma cascade against depression caused by neuropathic pain in rats. J. Basic Clin. Physiol. Pharmacol..

[16] Tufano M, Pinna G (2020). Is there a future for PPARs in the treatment of neuropsychiatric disorders?. Molecules.

[17] Lim J, Kim HI, Bang Y, Choi HJ (2021). Peroxisome proliferator-activated receptor gamma: A novel therapeutic target for cognitive impairment and mood disorders that functions via the regulation of adult neurogenesis. Arch. Pharm. Res..

[18] Motallebi M (2022). Naringenin: A potential flavonoid phytochemical for cancer therapy. Life Sci..

[19] Li S (2019). Naringenin improves insulin sensitivity in gestational diabetes mellitus mice through AMPK. Nutr. Diabetes.

[20] Kandhare AD, Ghosh P, Bodhankar SL (2014). Naringin, a flavanone glycoside, promotes angiogenesis and inhibits endothelial apoptosis through modulation of inflammatory and growth factor expression in diabetic foot ulcer in rats. Chem. Biol. Interact..

[21] Rehman K (2020). Naringenin downregulates inflammation-mediated nitric oxide overproduction and potentiates endogenous antioxidant status during hyperglycemia. J. Food Biochem..

[22] Singh AK (2018). Isolated mangiferin and naringenin exert antidiabetic effect via PPARgamma/GLUT4 dual agonistic action with strong metabolic regulation. Chem. Biol. Interact..

[23] Guadarrama-Escobar OR (2023). Chitosan nanoparticles as oral drug carriers. Int. J. Mol. Sci..

[24] Bai Y (2020). Pharmacokinetics and metabolism of naringin and active metabolite naringenin in rats, dogs, humans, and the differences between species. Front. Pharmacol..

[25] Smruthi MR, Nallamuthu I, Anand T (2022). A comparative study of optimized naringenin nanoformulations using nano-carriers (PLA/PVA and zein/pectin) for improvement of bioavailability. Food Chem..

[26] Patra JK (2018). Nano based drug delivery systems: recent developments and future prospects. J. Nanobiotechnol..

[27] Tan SLJ, Billa N (2021). Improved bioavailability of poorly soluble drugs through gastrointestinal muco-adhesion of lipid nanoparticles. Pharmaceutics.

[28] Fang RH, Aryal S, Hu CM, Zhang L (2010). Quick synthesis of lipid-polymer hybrid nanoparticles with low polydispersity using a single-step sonication method. Langmuir.

[29] Mukherjee A (2019). Lipid-polymer hybrid nanoparticles as a next-generation drug delivery platform: state of the art, emerging technologies, and perspectives. Int. J. Nanomed..

[30] Khan MM (2020). Folate targeted lipid chitosan hybrid nanoparticles for enhanced anti-tumor efficacy. Nanomed. Nanotechnol. Biol. Med..

[31] Abdulhakeem S, Arafa M, Elmeshad A (2021). Hybrid chitosan-lipid nanoparticles of green tea extract as natural anti-cellulite agent with superior in vivo potency: full synthesis and analysis. Drug Deliv..

[32] Cheba BA (2011). Chitin and chitosan: marine biopoly-mers with unique properties and versatile applications. Glob. J. Biotechnol. Biochem..

[33] Wang L (2014). In vitro and in vivo evaluation of chitosan graft glyceryl monooleate as peroral delivery carrier of enoxaparin. Int. J. Pharmaceut..

[34] Ou XM (2008). Simvastatin attenuates bleomycin-induced pulmonary fibrosis in mice. Chin Med J (Engl).

[35] Olugbemide AS (2021). Naringenin improves depressive- and anxiety-like behaviors in mice exposed to repeated hypoxic stress through modulation of oxido-inflammatory mediators and NF-kB/BDNF expressions. Brain Res. Bull..

[36] Bansal Y (2018). Naringenin protects against oxido-inflammatory aberrations and altered tryptophan metabolism in olfactory bulbectomized-mice model of depression. Toxicol. Appl. Pharmacol..

[37] Tayyab M (2019). Antidepressant and neuroprotective effects of naringenin via sonic hedgehog-GLI1 cell signaling pathway in a rat model of chronic unpredictable mild stress. Neuromol. Med..

[38] Hamdi M (2020). An integrated vitamin E-coated polymer hybrid nanoplatform: A lucrative option for an enhanced in vitro macrophage retention for an anti-hepatitis B therapeutic prospect. PLoS One.

[39] Dong W (2018). Chitosan based polymer-lipid hybrid nanoparticles for oral delivery of enoxaparin. Int. J. Pharm..

[40] Wang L (2014). In vitro and in vivo evaluation of chitosan graft glyceryl monooleate as peroral delivery carrier of enoxaparin. Int. J. Pharm..

[41] Ji P (2016). Naringenin-loaded solid lipid nanoparticles: preparation, controlled delivery, cellular uptake, and pulmonary pharmacokinetics. Drug Des. Devel. Ther..

[42] Souto EB, Mehnert W, Muller RH (2006). Polymorphic behaviour of Compritol888 ATO as bulk lipid and as SLN and NLC. J. Microencapsul..

[43] Thapa RK, Yoo BK (2014). Evaluation of the effect of tacrolimus-loaded liquid crystalline nanoparticles on psoriasis-like skin inflammation. J. Dermatol. Treat.

[44] Yang SC (1999). Body distribution in mice of intravenously injected camptothecin solid lipid nanoparticles and targeting effect on brain. J Control Release.

[45] Maity S, Mukhopadhyay P, Kundu PP, Chakraborti AS (2017). Alginate coated chitosan core-shell nanoparticles for efficient oral delivery of naringenin in diabetic animals-An in vitro and in vivo approach. Carbohydr. Polym..

[46] Vanti G (2019). Development and percutaneous permeation study of escinosomes, escin-based nanovesicles loaded with berberine chloride. Pharmaceutics.

[47] AbouSamra MM, Afifi SM, Galal AF, Kamel R (2023). Rutin-loaded Phyto-Sterosomes as a potential approach for the treatment of hepatocellular carcinoma: In-vitro and in-vivo studies. J. Drug Deliv. Sci. Technol..

[48] Soleimanian Y (2020). beta-sitosterol loaded nanostructured lipid carrier: Physical and oxidative stability, in vitro simulated digestion and hypocholesterolemic activity. Pharmaceutics.

[49] Tubesha Z, Imam MU, Mahmud R, Ismail M (2013). Study on the potential toxicity of a thymoquinone-rich fraction nanoemulsion in Sprague Dawley rats. Molecules.

[50] Khan MF, Mathur A, Pandey VK, Kakkar P (2022). Endoplasmic reticulum stress-dependent activation of TRB3-FoxO1 signaling pathway exacerbates hyperglycemic nephrotoxicity: Protection accorded by Naringenin. Eur. J. Pharmacol..

[51] Büyüktuncel E (2017). Fast determination of naringin and hesperidin in natural and commercial citrus juices by HPLC method. Asian J. Chem..

[52] El-Marasy SA (2023). Chrysin loaded nanovesicles ameliorated diabetic peripheral neuropathy. Role of NGF/AKT/GSK-3beta pathway. Chem. Biol. Interact..

[53] Moustafa PE (2018). Extracellular matrix remodeling and modulation of inflammation and oxidative stress by sulforaphane in experimental diabetic peripheral neuropathy. Inflammation.

[54] Moustafa PE (2018). Liraglutide ameliorated peripheral neuropathy in diabetic rats: Involvement of oxidative stress, inflammation and extracellular matrix remodeling. J. Neurochem..

[55] Elshazly SM, Abd El Motteleb DM, Ibrahim I (2018). Hesperidin protects against stress induced gastric ulcer through regulation of peroxisome proliferator activator receptor gamma in diabetic rats. Chem. Biol. Interact..

[56] Can OD, Ozturk Y, Ozkay UD (2011). Effects of insulin and St. John's Wort treatments on anxiety, locomotory activity, depression, and active learning parameters of streptozotocin-diabetic rats. Planta Med..

[57] El-Marasy SA (2021). Anti-depressant effect of cerebrolysin in reserpine-induced depression in rats: Behavioral, biochemical, molecular and immunohistochemical evidence. Chem Biol Interact.

[58] Tomaz LM (2016). GLUT2 proteins and PPARgamma transcripts levels are increased in liver of ovariectomized rats: reversal effects of resistance training. J. Exerc. Nutrit. Biochem..

[59] Khan MM (2020). Folate targeted lipid chitosan hybrid nanoparticles for enhanced anti-tumor efficacy. Nanomedicine.

[60] Livak KJ, Schmittgen TD (2001). Analysis of relative gene expression data using real-time quantitative PCR and the 2(-Delta Delta C(T)) method. Methods.

[61] Pagel P, Blome J, Wolf HU (2000). High-performance liquid chromatographic separation and measurement of various biogenic compounds possibly involved in the pathomechanism of Parkinson's disease. J. Chromatogr. B Biomed. Sci. Appl..

[62] Dave V (2017). Lipid-polymer hybrid nanoparticles: Development & statistical optimization of norfloxacin for topical drug delivery system. Bioact. Mater..

[63] Kulkarni AR, Soppimath KS, Aminabhavi TM, Rudzinski WE (2001). In-vitro release kinetics of cefadroxil-loaded sodium alginate interpenetrating network beads. Eur. J. Pharm. Biopharm..

[64] Aulton ME, Taylor K (2013). Aulton's Pharmaceutics: The Design and Manufacture of Medicines.

[65] AbouSamra MM, Salama AH (2017). Enhancement of the topical tolnaftate delivery for the treatment of tinea pedis via provesicular gel systems. J. Liposome Res..

[66] Chen M, Liu X, Fahr A (2011). Skin penetration and deposition of carboxyfluorescein and temoporfin from different lipid vesicular systems: In vitro study with finite and infinite dosage application. Int. J. Pharm..

[67] Hafner A, Lovric J, Pepic I, Filipovic-Grcic J (2011). Lecithin/chitosan nanoparticles for transdermal delivery of melatonin. J. Microencapsul..

[68] Sun Y (2009). The effect of chitosan molecular weight on the characteristics of spray-dried methotrexate-loaded chitosan microspheres for nasal administration. Drug Dev. Ind. Pharm..

[69] Tahir N (2019). Lipid-polymer hybrid nanoparticles for controlled delivery of hydrophilic and lipophilic doxorubicin for breast cancer therapy. Int. J. Nanomed..

[70] Tortorici S (2022). Nanostructured lipid carriers of essential oils as potential tools for the sustainable control of insect pests. Ind. Crops Prod..

[71] Kamel R, AbouSamra MM, Afifi SM, Galal AF (2022). Phyto-emulsomes as a novel nano-carrier for morine hydrate to combat leukemia: In vitro and pharmacokinetic study. J. Drug Deliv. Sci. Technol..

[72] Basha M, AbouSamra MM, Awad GA, Mansy SS (2018). A potential antibacterial wound dressing of cefadroxil chitosan nanoparticles in situ gel: Fabrication, in vitro optimization and in vivo evaluation. Int. J. Pharm..

[73] Saravanakumar G (2009). Hydrotropic oligomer-conjugated glycol chitosan as a carrier of paclitaxel: synthesis, characterization, and in vivo biodistribution. J. Control Release.

[74] EmadEldeeb A, Salah S, Ghorab M (2019). Proniosomal gel-derived niosomes: an approach to sustain and improve the ocular delivery of brimonidine tartrate; formulation, in-vitro characterization, and in-vivo pharmacodynamic study. Drug Deliv..

[75] Ramaswamy S (2017). Formulation and characterization of chitosan encapsulated Phytoconstituents of curcumin and Rutin nanoparticles. Int. J. Biol. Macromol..

[76] Gorantla S (2022). Recent advances in nanocarriers for nutrient delivery. Drug Deliv. Transl. Res..

[77] Khan MI (2022). Recent progress in nanostructured smart drug delivery systems for cancer therapy: A review. ACS Appl. Bio. Mater..

[78] Wang J (2023). Simultaneously inhibiting P-gp Efflux and drug recrystallization enhanced the oral bioavailability of nintedanib. Curr. Pharm. Biotechnol..

[79] Banerjee S, Pillai J (2019). Solid lipid matrix mediated nanoarchitectonics for improved oral bioavailability of drugs. Expert Opin. Drug Metab. Toxicol..

[80] Scioli Montoto S, Muraca G, Ruiz ME (2020). Solid lipid nanoparticles for drug delivery: Pharmacological and biopharmaceutical aspects. Front. Mol. Biosci..

[81] Abu Elfadl A, Boughdady M, Meshali M (2021). New Peceol/Span 60 niosomes coated with chitosan for candesartan cilexetil: Perspective increase in absolute bioavailability in rats. Int. J. Nanomed..

[82] Gagliardi A (2020). Design and characterization of glyceryl monooleate-nanostructures containing doxorubicin hydrochloride. Pharmaceutics.

[83] Kumar, P., K.R. Gajbhiye, K.M. Paknikar, &V. Gajbhiye, *Chapter 1 - Current Status and Future Challenges of Various Polymers as Cancer Therapeutics*, in *Polymeric Nanoparticles as a Promising Tool for Anti-cancer Therapeutics*, P. Kesharwani, K.M. Paknikar, and V. Gajbhiye, Editors. 2019, Academic Press. p. 1–20.10.1016/B978-0-12-816963-6.00001-7

[84] Fries GR, Saldana VA, Finnstein J, Rein T (2023). Molecular pathways of major depressive disorder converge on the synapse. Mol. Psychiat..

[85] Rahmani G (2020). Garlic (Allium sativum) improves anxiety- and depressive-related behaviors and brain oxidative stress in diabetic rats. Arch. Physiol. Biochem..

[86] Bampi SR (2020). Depression-like behavior, hyperglycemia, oxidative stress, and neuroinflammation presented in diabetic mice are reversed by the administration of 1-methyl-3-(phenylselanyl)-1H-indole. J. Psychiatr. Res..

[87] Singh P (2020). Naringenin ameliorates diabetic neuropathic pain by modulation of oxidative-nitrosative stress, cytokines and MMP-9 levels. Food Funct..

[88] Al-Dosari DI (2017). Flavonoid Naringenin attenuates oxidative stress, apoptosis and improves neurotrophic effects in the diabetic rat retina. Nutrients.

[89] Haider S (2020). Naringenin protects AlCl3/D-galactose induced neurotoxicity in rat model of AD via attenuation of acetylcholinesterase levels and inhibition of oxidative stress. PLoS One.

[90] Zimath PL (2021). Myrsinoic acid B from Myrsine coriacea reverses depressive-like behavior and brain oxidative stress in streptozotocin-diabetic rats. Chem. Biol. Interact..

[91] Wang YL (2021). Catalpol ameliorates depressive-like behaviors in CUMS mice via oxidative stress-mediated NLRP3 inflammasome and neuroinflammation. Transl. Psychiat..

[92] Hannestad J, DellaGioia N, Bloch M (2011). The effect of antidepressant medication treatment on serum levels of inflammatory cytokines: a meta-analysis. Neuropsychopharmacology.

[93] Song MT (2018). Astragaloside IV ameliorates neuroinflammation-induced depressive-like behaviors in mice via the PPARgamma/NF-kappaB/NLRP3 inflammasome axis. Acta Pharmacol. Sin..

[94] Yang W (2014). Effect of naringenin on brain insulin signaling and cognitive functions in ICV-STZ induced dementia model of rats. Neurol. Sci..

[95] Raza SS (2013). Neuroprotective effect of naringenin is mediated through suppression of NF-kappaB signaling pathway in experimental stroke. Neuroscience.

