# Supplementary material for: Anti-depressant effect of Naringenin-loaded hybridized nanoparticles in diabetic rats via PPARγ/NLRP3 pathway
Source: Sci Rep. 2024 Jun 12;14:13559. doi: 10.1038/s41598-024-62676-x (PMC11169681; doi:10.1038/s41598-024-62676-x)
Supplement: Supplementary file 1 — Supplementary Information. [file 41598_2024_62676_MOESM1_ESM.docx]

**S1: Limit of detection (LOD) for Naringenin (NAR) separated by reversed-phase liquid chromatography with UV detector**
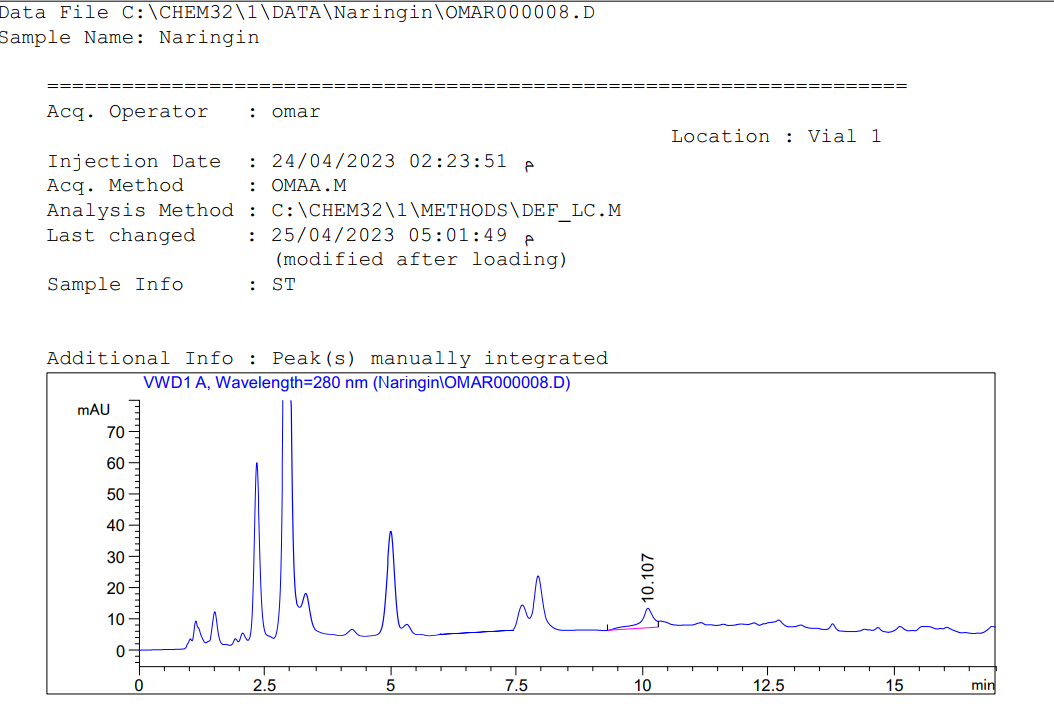


The chromatogram illustrates the LOD of NAR at retention time 10.107

| 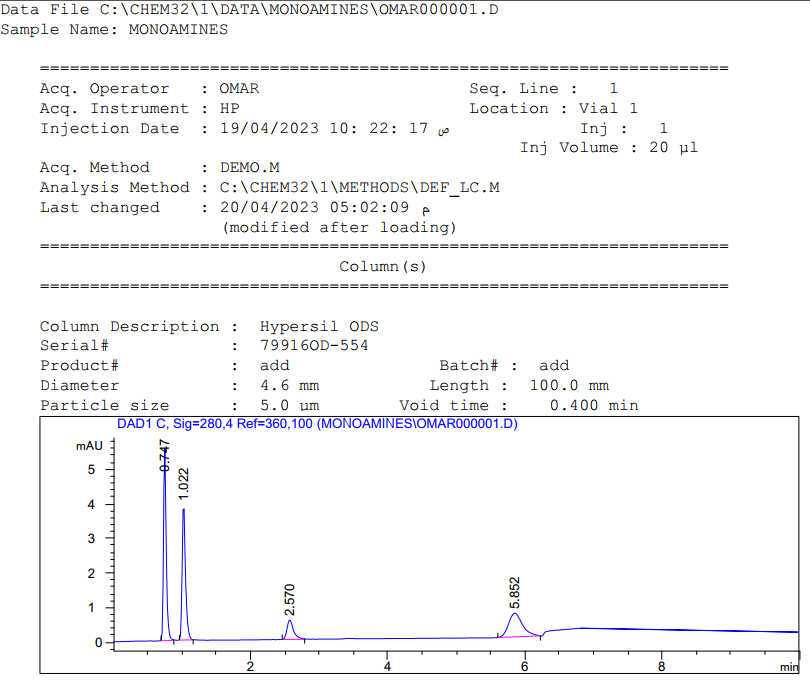 |
| --- |
| The chromatogram illustrates the LOD of NE, DA, 5-HT at retention time 1.023, 2.570, and  5.852, respectively. |

**S2: Limit of detection (LOD) for monoamines separated by reversed-phase liquid chromatography with UV detector.**
